# Expression of NEAT1 induced by influenza virus infection is regulated by activated STAT3 and contributes to STAT3-mediated antiviral immunity

**DOI:** 10.3389/fimmu.2025.1693884

**Published:** 2025-10-23

**Authors:** Rongrong Gu, Erying Xu, Jingjie Hong, Liqing Fan, Shasha Liu, Ji-Long Chen

**Affiliations:** ^1^ Key Laboratory of Animal Pathogen Infection and Immunology of Fujian Province, College of Animal Sciences, Fujian Agriculture and Forestry University, Fuzhou, China; ^2^ Key Laboratory of Fujian-Taiwan Animal Pathogen Biology, College of Animal Sciences, Fujian Agriculture and Forestry University, Fuzhou, China; ^3^ Joint Laboratory of Animal Pathogen Prevention and Control of Fujian-Nepal, College of Animal Sciences, Fujian Agriculture and Forestry University, Fuzhou, China; ^4^ Institute of Plateau Ecology, Xizang Agricultural and Animal Husbandry University, Linzhi, China

**Keywords:** lncRNA, NEAT1, STAT3, influenza virus, innate immunity

## Abstract

The transcription factor STAT3 is integral to the immune response during viral infections, while long non-coding RNAs (lncRNAs) are actively implicated in the modulation of viral pathogenesis. However, the relationship between STAT3 and lncRNAs during viral infection is poorly understood. Here, we observed that robust expression of NEAT1, an important lncRNA, was induced by infections with influenza A virus (IAV) and several other viruses, but the virus-induced NEAT1 expression was significantly suppressed by inactivation of STAT3 both *in vitro* and *in vivo*. Furthermore, we identified that expression of NEAT1 was regulated via MDA5 and TLR3 signaling pathways involving NF-κB, IL-6, and IFN-β during IAV infection. Disruption of NEAT1 expression markedly facilitated the replication of IAV, whereas overexpression of NEAT1 attenuated the viral replication. NEAT1 knockout mice were further employed and showed that deficiency of NEAT1 significantly enhanced the IAV replication and virulence in the animals. Importantly, we found that activation of STAT3 by innate immune signaling inhibited IAV infection through upregulating the expression of NEAT1, and NEAT1 promoted the production of several vital antiviral molecules including interferons (IFNs) to suppress the viral replication. Moreover, our experiments exhibited that NEAT1 contributed to activation of TBK1 during the IAV infection. Together, these results reveal that NEAT1 functions downstream of STAT3, acting as a regulator of STAT3-mediated immunity by activating TBK1 and thereby enhancing antiviral responses.

## Introduction

Influenza A virus (IAV) is one of the major pathogens of zoonotic diseases, infecting birds and mammals such as pigs, horses, martens, seals, and humans (1, 2). The host innate immune response to viral infections encompasses multiple signaling pathways, with the Janus kinase/signal transducer and activator of transcription (JAK-STAT) pathway playing a key role (3, 4). Following the invasion of host by IAV, its pathogen-associated molecular patterns (PAMPs) are recognized by pattern recognition receptors (PRRs), including RIG-I, MDA5, and TLR3 (5, 6). In turn, the signal is transmitted to downstream adaptor proteins MAVS or TRIF (7), which recruits and activates corresponding transcription factors such as IRF3/7 and NF-κB that govern the expression of a series of cytokines (8). These cytokines bind to their receptors, activate JAK-STAT and other signaling pathways (9, 10), and thereby induce the expression of various interferon-stimulated genes (ISGs) and other effector genes (11).

STAT3 is an important transcription factor involved in the regulation of a variety of biological processes, and abnormally activated in diversified cancers (12–15). The interaction between STAT3 and viruses is extremely complex (16). In response to viral infections, STAT3 can be activated or inhibited (17). For example, Ebola virus (EBOV) inhibited STAT3 phosphorylation or nucleation (18). However, IAV infection significantly enhanced STAT3 phosphorylation at the site of Y705, and point mutation of Y705 in mice (STAT3^Y705F/+^) obviously impaired host antiviral immunity (19). The viral protein ORF3A of SARS-CoV-2 elevated the protein level of tripartite motif-containing protein 59 (TRIM59), and hence inhibited the dephosphorylation of STAT3, leading to persistent STAT3 activation (20). On the other hand, it has been shown that STAT3 could promote some viral infections. For instance, STAT3 could promote hepatitis E virus (HEV) replication, as evidenced by significantly reduced expression of HEV ORF2 protein caused by STAT3 inactivation (21). In addition, grass carp reovirus (GCRV) VP7 protein promoted the activation of STAT3, and ultimately assisted virus to complete the invasion (22).

It has been reported that STAT3 can interact with long non-coding RNAs (lncRNAs) to regulate the occurrence and development of cancer (23). For instance, STAT3 could specifically promote the transcription of lncRNA HOXD-AS1 and prevented the degradation of SOX4, thus promoting the metastasis of hepatocellular carcinoma (HCC) (24). LncRNA LINC00908 encodes a 60 aa polypeptide ASRPS that inhibited tumor growth by down-regulating the phosphorylation of STAT3, resulting in decreased expression of vascular endothelial growth factor (VEGF) (25). LncRNA-H19 inhibited STAT3 signaling pathway by reducing the expression level of miRNA-675-3p, leading to an increased incidence of pancreatic cancer (26). However, little information is available about interaction between STAT3 and lncRNAs during viral infection.

The genome of higher mammals exhibits extensive transcription of lncRNAs with diverse biological functions. Typically, lncRNAs are transcribed by RNA polymerase II or III and undergo post-transcriptional modifications including 5’ capping and 3’ polyadenylation (27, 28). LncRNAs can be broadly categorized into five types based on their genomic positions relative to neighboring protein-coding genes: sense, antisense, bidirectional, intronic, and intergenic lncRNAs (29). An increasing body of evidence demonstrates that lncRNAs play crucial regulatory roles in diverse biological processes such as growth, development, viral infection, immune response, and disease occurrence (30, 31). Notably, previous studies have highlighted the involvement of several lncRNAs in the regulation of IAV infection and its associated pathogenesis (32). *In vivo* experiments demonstrated that lncRNA AVAN induced the secretion of IFN-α and IFN-β, and expression of ISGs, which inhibited the replication of IAV and thereby improved the survival rate of mice (33). IAV-induced lncRNA IFITM4P positively regulated the expression of other IFITM family members through competitive binding of miR-24-3p and miR-122-5p (34). Primate-specific lncRNA CHROMR induced by IAV and SARS-CoV-2 infection could coordinate the expression of some ISGs (35). The MIR155HG gene codes both lncRNA-155 and miRNA-155 implicated in host-virus interaction (36). LncRNA-155 promoted the production of IFN-β by regulating the phosphorylation of IRF3, while miRNA-155-5p enhanced the antiviral response by promoting the activation of STAT1 (37). In addition, Cao et al. discovered that IAV-induced lncRNA USP30-AS1 functions as an interferon-stimulating gene (38). Although progress has been made in understanding the role of lncRNAs in IAV infection, functional involvement and underlying mechanisms of a large number of lncRNAs in IAV pathogenesis remain to be determined.

NEAT1 is one of the lncRNAs with multiple biological activities. An increasing number of studies reveal that NEAT1 is implicated in the regulation of cell cycle, proliferation, apoptosis, and migration of tumor cells (39, 40). However, functional involvement of NEAT1 in the IAV infection and pathogenesis remains elusive. In this study, we observed that robust expression of NEAT1 was induced by infections with IAV and several other viruses. Interestingly, inactivated-STAT3 caused significantly reduced expression of NEAT1 during IAV infection both *in vitro* and *in vivo*. *In vitro* experiments displayed that disruption of NEAT1 expression markedly facilitated the replication of IAV, whereas overexpression of NEAT1 attenuated the viral replication. Similarly, deficiency of mouse NEAT1 significantly increased IAV replication and virulence in the animals. Furthermore, activated-STAT3 impaired IAV replication by upregulating the expression of NEAT1. Additionally, we found that NEAT1 contributed to the activation of TBK1 during the IAV infection. Together, these data reveal that IAV-induced NEAT1 is regulated by activated STAT3 and contributes to STAT3-mediated antiviral immunity.

## Materials and methods

### Cell lines and cell culture

The A549, 293T, K562, HeLa, NIH/3T3, L929, and MDCK cell lines were purchased from American Type Culture Collection (Manassas, VA, USA). Cells were cultured in Dulbecco’s modified Eagle’s medium (DMEM) or RPMI 1640 (Gibco, Grand Island, NY, USA) supplemented with 10% fetal bovine serum (Gibco, Grand Island, NY, USA) and 100 U/mL penicillin-streptomycin (Beyotime Biotechnology, Shanghai, China) at 37 °C under a humidified 5% CO_2_ atmosphere.

### Viruses and viral infection

The viruses used in this study were propagated as follows: Influenza virus A/WSN/33 (H1N1) (WSN), Influenza virus A/PR/8/34 (H1N1) (PR8), Influenza virus A/CA/04/09 (H1N1) (CA04), and Sendai virus (SeV) in specific-pathogen-free (SPF) chicken embryos; Muscovy duck reovirus (MDRV) in duckembryo fibroblast cells; Pseudorabies virus (PRV) in PK-15 cells. PR8, CA04, SeV, and PRV were used to infect A549 cells. MDRV was employed to infect 293T cells. Cells were incubated with virus for 1 h and cultured in DMEM for the indicated times.

### Cell stimulation

Reagents, including poly(I:C), dimethyl sulfoxide (DMSO) (Sigma-Aldrich, Germany), tocilizumab (Selleck, USA), and recombinant human IFN-β and IL-6 (PeproTech, USA) were purchased, and cells were treated following the manufacturer’s instructions.

### RNA preparation, RT-PCR, and quantitative real-time PCR

Total RNA was extracted using TRIzol reagent (TIANGEN, China) according to the manufacturer’s instructions. cDNA was synthesized by a HiScript III 1st Strand cDNA Synthesis Kit (Vazyme, Nanjing, China). cDNA synthesis was followed by PCR using Taq DNA polymerase (GenStar, Beijing, China) or SYBR Green Master Mix (Vazyme, Nanjing, China) for quantitative real-time PCR. Primers used are shown in **Table 1**. β‐actin was chosen as a reference housekeeping gene for internal standardization. For quantification, the 2−ΔΔCt method was used to calculate the relative RNA levels against β‐actin.

**Table 1:** Sequences of primers used in this study.

Gene name	Sequences
NEAT1 (NEAT1_1+NEAT1_2) (human)	Forward: TGCTGCGTATGCAAGTCTGA Reverse: GAGAACCAAAGGGAGGGGTG
NEAT1_2 (human)	Forward: GATCTTTTCCACCCCAAGAGTACATAA Reverse: CTCACACAAACACAGATTCCACAAC
mNEAT1 (NEAT1_1+NEAT1_2) (mouse)	Forward: AGGAGAAGCGGGGCTAAGTA Reverse: TAGGACACTGCCCCCATGTA
mNEAT1_2 (mouse)	Forward: CCTTGAGCCTGCAGACAAGA Reverse: TGAGACTGGCCTGGGACATA
IAV-NP	Forward: TCAAACGTGGGATCAATG Reverse: GTGCAGACCGTGCTAGAA
PRV-gE	Forward: CTTCCACTCGCAGCTCTTCT Reverse: TAGATGCAGGGCTCGTACAC
MDRV-P10	Forward: ATGGCTGACGCTTTTGAAGT Reverse: TAGTTAGATCTCGAGAGCCCG
SeV-NP	Forward: ATAAGTCGGGAGGAGGTGCT Reverse: GTTGACCCTGGAAGAGTGGG
β-actin (human/mouse)	Forward: GCTGCCTCAACACCTCAACCC Reverse: GTCCCTCACCCTCCCAAAAG
IL-6 (human)	Forward: AATGAGGAGACTTGCCTGGTG Reverse: TGAGGTGCCCATGCTACATT
IL-6 (mouse)	Forward: GGGACTGATGCTGGTGACAA Reverse: CGCACTAGGTTTGCCGAGTA
IFN-α (human)	Forward: CCTGATGAATGCGGACTCCA Reverse: ATAGCAGGGGTGAGAGTCTT
IFN-α (mouse)	Forward: AGGACTTTGGATTCCCGCAG Reverse: ATCAGACAGCCTTGCAGGTC
IFN-β (human)	Forward: GCTCTCCTGTTGTGCTTCTCCAC Reverse: CAATAGTCTCATTCCAGCCAGTGC
IFN-β (mouse)	Forward: GGTCCGAGCAGAGATCTTCA Reverse: CACTACCAGTCCCAGAGTCC
ISG15 (human)	Forward: CTCTGAGCATCCTGGTGAGGAA Reverse: AAGGTCAGCCAGAACAGGTCGT
MX1 (human)	Forward: GACATTCGGCTGTTTACC Reverse: GCGGTTCTGTGGAGGTTA
IFIT2 (human)	Forward: AGCGAAGGTGTGCTTTGAGA Reverse: GAGGGTCAATGGCGTTCTGA

### Antibodies and Western blotting

Cells were lysed with radio immunoprecipitation assay (RIPA) buffer supplemented with protease inhibitors. Immunoprecipitates were washed three times with lysis buffer, then separated by SDS-PAGE, and transferred onto nitrocellulose membrane, and probed with antibodies as indicated.

### Hemagglutinin assay and plaque-forming assay

MDCK cells were infected with serial dilutions of the viruses. After an incubation period, cells were washed with PBS and overlaid with DMEM containing 1.5% low melting point agarose (Promega, Madison, WI, USA) and 2 μg/mL TPCK (tolylsulfonyl phenylalanyl chloromethyl ketone)-treated trypsin (Sigma-Aldrich, St. Louis, MO, USA). After 72 h of incubation at 37 °C, plaques were stained and counted. For hemagglutinin (HA) assay, the supernatants were diluted with PBS and mixed with an equal volume of 0.5% chicken erythrocytes. Then, viral titers were counted from the highest dilution factors that produced a positive reading (41).

### Dual-luciferase reporter assay

The 293T cells were seeded in 24-well culture plates. IFN-β-Luc was co-transfected with pRL-TK (MiaoLing Plasmid Platform, Wuhan, China), along with indicated plasmids using Lipo8000 (Beyotime Institute of Biotechnology, Jiangsu, China). At 24 h post-transfection, luciferase activity was measured using the dual-luciferase reporter assay system (Promega, WI, USA). Luciferase activity was normalized to that of Renilla luciferase activity as previously described (42).

### Generation of stable cell lines

Cells stably expressing NEAT1_1 or empty vector (EV) were generated by infecting A549 cells with lentiviruses encoding these genes in pLVX3 vector. ShRNAs were designed for knockdown of human NEAT1, NEAT1_2, RIG-I, MDA5, TLR3, NF-κB, IRF3, IRF7, and STAT3. The sh-RNA sequences are shown in **Table 2**. And other sh-RNA or overexpressing sequences involved in this study were as previously described (43).

**Table 2:** Sequences of shRNAs used in this study.

shRNAs	Sequences (5’-3’)
sh-1#-NEAT1	GGAAGGCAGGGAGAGGTAGAA
sh-2#-NEAT1	GTGAGAAGTTGCTTAGAAACT
sh-NEAT1_2	GGGTAAATCTCAATCTTAATC
sh-RIG-I	GCAGAGAAATTGGTGGAATGC
sh-MDA5	CCAACAAAGAAGCAGTGTATA
sh-IRF3	CATTGTAGATCTGATTACCTTC
sh-IRF7	GCCTCTATGACGACATCGAGT
sh-NF-κB	GCGACAAGGTGCAGAAAGA
sh-STAT3	GCAGCAGCTGAACAACATG
sh-Luciferase	CTTACGCTGAGTACTTCGA

### Animal experiments

Wild type (WT) C57BL/6J mice or WT BALB/c mice (6 weeks old, 18 to 21g) were obtained from Shanghai SLAC Laboratory Animal (Shanghai, China). IFNAR1 knockout (IFNAR1^-/-^) mice on C57BL/6J background and STAT3^Y705F/+^ mice on BALB/c background were employed as previously described (19). NEAT1 knockout (NEAT1^-/-^) mice on C57BL/6J background were purchased from Gem Pharmatech Co. Itd (strain ID: T011757). All mice were housed and bred in the animal facility at Fujian Agriculture and Forestry University, under specific pathogen free conditions. The animals were fed with standard food which was available *ad libitum*. Mice were intranasally inoculated with IAV. Once a mouse lost above 25% of its original weight, it was sacrificed. Mice were sacrificed by CO_2_ gassing. All animal experiments were reviewed and approved by the Regulation of College of Animal Sciences, Fujian Agriculture and Forestry University of Research Ethics Committee. Efforts were made to minimize animal suffering.

### Statistical analysis

Comparison between groups was made using Student’s t test. Data represent the mean ± SD from three independent experiments. Differences were considered statistically significant with *p* < 0.05.

## Results

### Activated STAT3 is required for IAV-induced expression of NEAT1 *in vitro* and *in vivo*

**Figure 1:**
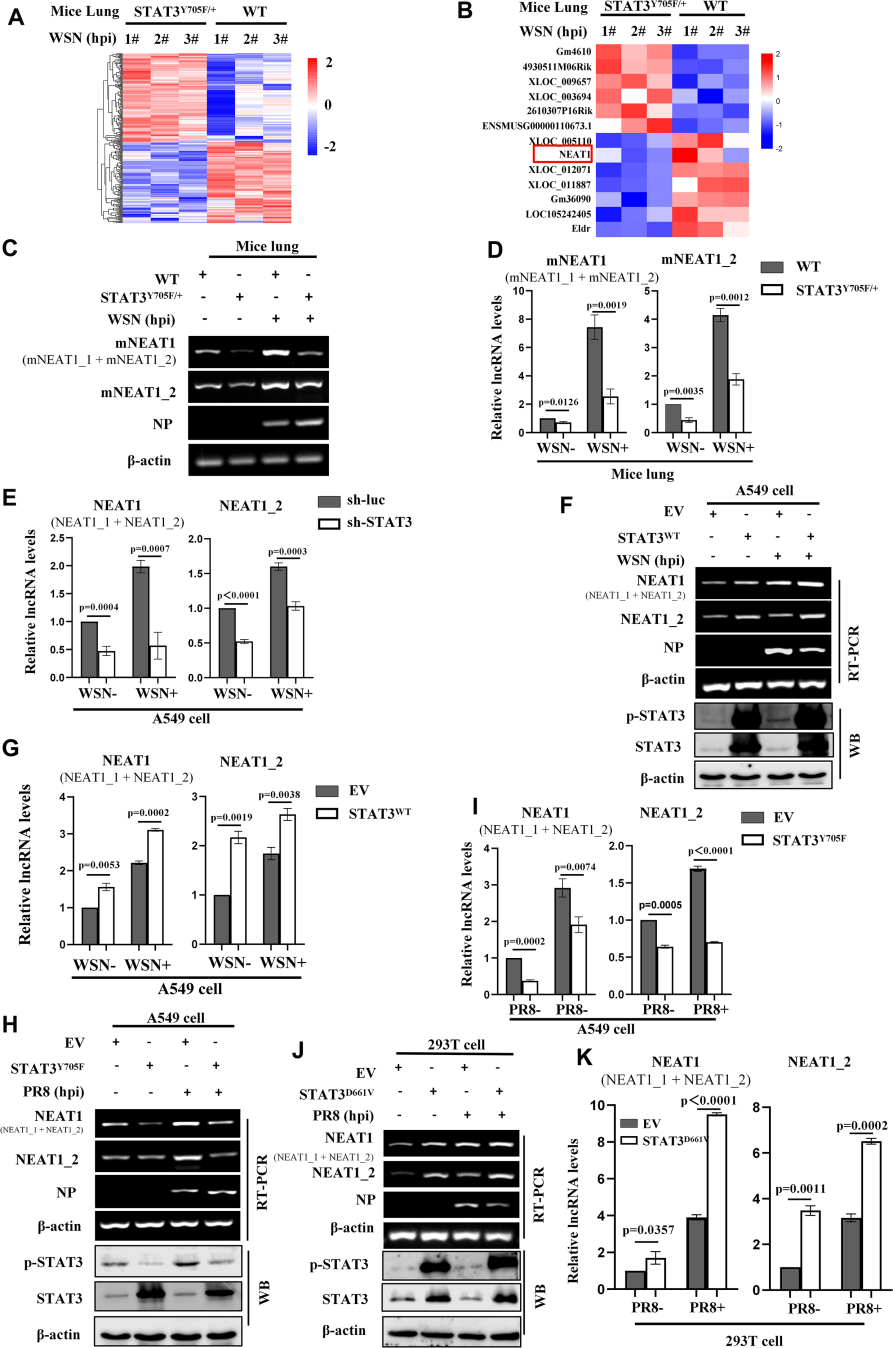
Activated STAT3 is required for IAV-induced expression of NEAT1 *in vitro* and *in vivo*. **(A, B)** 6 weeks-old BALB/c wild type (WT) mice or STAT3^Y705F/+^ mice on BALB/c background were infected intranasally with WSN (5×10^4^ PFU/mL) for 24 **(H)** Then mice were sacrificed and the lungs were collected. The differentially expressed lncRNAs were detected by RNA-Seq (fold change >2, *P* < 0.05) **(A)**. The RNA quantitation is scaled log_2_ data in heat maps **(B)**. **(C, D)** The expression of mouse NEAT1 (mNEAT1 and mNEAT1_2) in WSN-infected or mock-infected lungs of STAT3^Y705F/+^ mice were detected by RT-PCR **(C)** and quantitative real-time PCR **(D)**. Data are represented as mean ± SD from three independent experiments. **(E)** A549 cells expressing specific shRNAs targeting STAT3 and luciferase (sh-luc), were infected with or without WSN (MOI = 1) for 16 **(H)** The expression of human NEAT1 (NEAT1 and NEAT1_2) was detected by quantitative real-time PCR. Data are represented as mean ± SD from three independent experiments. **(F-I)** A549 cells overexpressing Flag-STAT3^WT^ (STAT3^WT^) **(F, G)**, Flag-STAT3^Y705F^ (STAT3^Y705F^) **(H, I)**, or empty vector (EV) were infected with or without IAV (MOI = 1) for 16 **(H)** The expression of human NEAT1 (NEAT1 and NEAT1_2) was detected by RT-PCR **(F, H)** and quantitative real-time PCR **(G, H)**. Data are represented as mean ± SD from three independent experiments. **(J, K)** 293T cells overexpressing Flag-STAT3^D661V^ (STAT3^D661V^) or empty vector (EV) were infected with or without PR8 (MOI = 1) for 16 h. The expression of human NEAT1 (NEAT1 and NEAT1_2) was detected by RT-PCR and quantitative real-time PCR **(J, K)**. Data are represented as mean ± SD from three independent experiments. See also **Supplementary Figure S1**.

Our previous studies reveal that activated STAT3 is involved in antiviral immunity against IAV infection (19), but the precise mechanism is unclear. In order to identify key lncRNAs implicated in the antiviral responses mediated by STAT3, RNA-seq analysis was performed on lung tissues of STAT3^Y705F/+^ and wild type (WT) mice infected with or without influenza virus A/WSN/33 (H1N1) (GEO: GSE213834). The data showed that expression of 177 lncRNAs was significantly changed, including 85 lncRNAs up-regulated, and 92 lncRNAs down-regulated in the lungs of IAV-infected STAT3^Y705F/+^ mice as compared to the WT control (fold change of>2; P<0.05) (**Figure 1A**). Among them, lncRNA NEAT1 was markedly down-regulated in the IAV-infected STAT3^Y705F/+^ mice (**Figure 1B**). NEAT1 is a lncRNA with multiple biological functions, and it has been reported that herpes simplex virus-1 (HSV-1) infection increases NEAT1 expression and paraspeckle formation in a STAT3-dependent manner (44). Thus, NEAT1 was selected for further study.

The mouse NEAT1 (mNEAT1) gene is located on chromosome 19 and encodes two transcriptional variants, NEAT1_1 (~ 3.2 kb) and NEAT1_2 (~ 21 kb) (**Supplementary Figure S1A**). The human NEAT1 gene is located on chromosome 11 and also encodes two transcripts, NEAT1_1 (~ 3.7 kb) and NEAT1_2 (~ 23 kb) (**Supplementary Figure S1B**). The differential expression of mouse NEAT1 was confirmed by RT-PCR and quantitative real-time PCR. We observed that mNEAT1 was significantly upregulated in mice lungs by the IAV infection, and disruption of STAT3 phosphorylation at Y705 remarkably decreased the NEAT1 expression (**Figures 1C, D**; **Supplementary Figure S1C**).

To further investigate the relationship between NEAT1 and STAT3 during IAV infection, *in vitro* systems were employed by using several cell lines. We found that silencing STAT3 clearly reduced the expression of NEAT1 in A549 cells (**Figure 1E**; **Supplementary Figure S1D**), whereas overexpression of wild-type STAT3 (STAT3^WT^) increased the expression of NEAT1 (**Figures 1F, G**). Moreover, the disruption of STAT3 Y705 (STAT3^Y705F^) resulted in significantly decreased expression of NEAT1 in A549 cells (**Figures 1H, I**). However, the expression of NEAT1 was highly induced in cells overexpressing the constitutively active mutant of STAT3 (STAT3^D661V^) (**Figures 1J, K**). Together, these results indicate that activated STAT3 regulates the NEAT1 expression during the IAV infection *in vitro* and *in vivo.*

### NEAT1 expression is significantly induced by infections with several viruses

**Figure 2:**
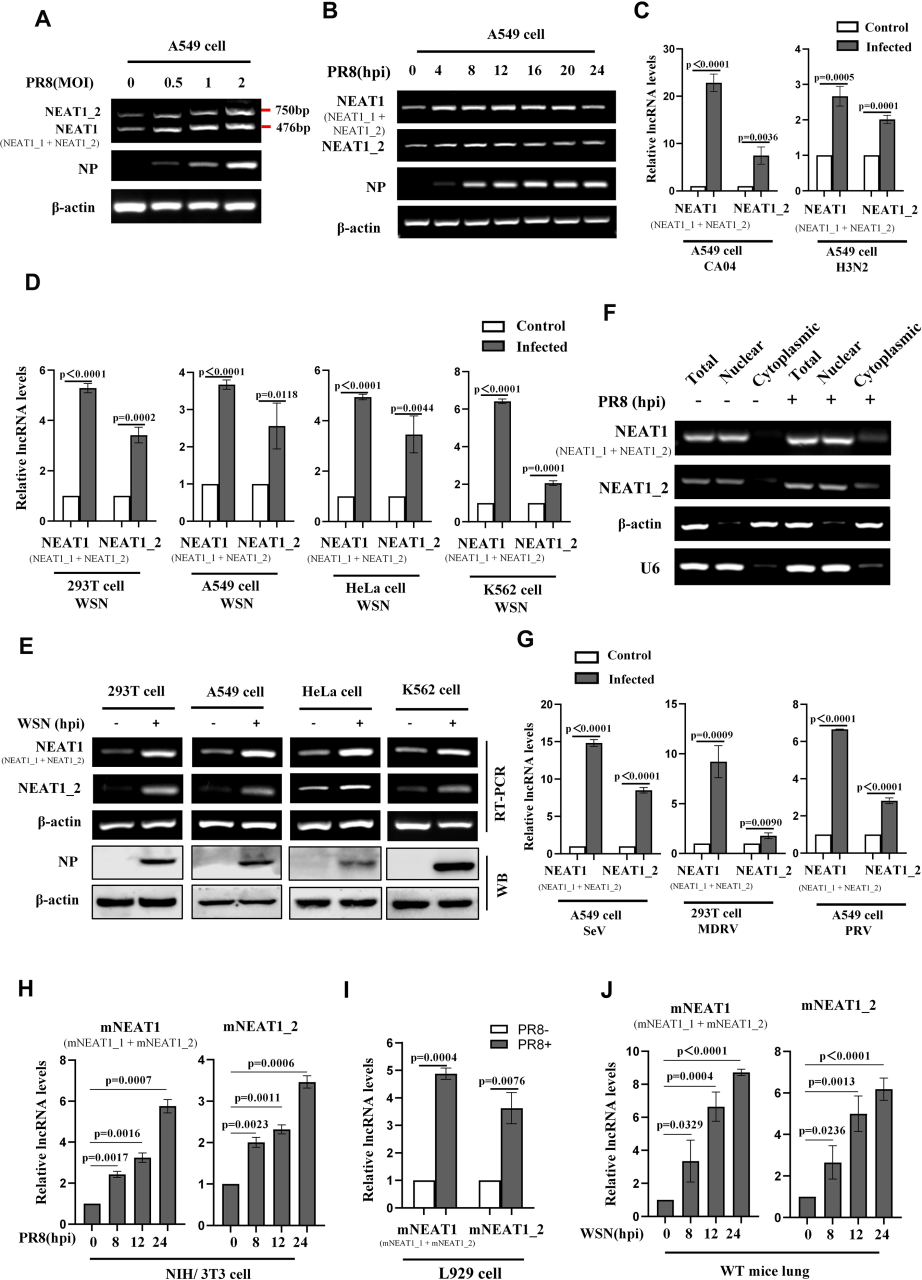
NEAT1 expression is significantly induced by infections with several viruses. **(A, B)** A549 cells were infected with or without PR8 at the indicated MOIs for 16 h **(A)** or at an MOI of 1 for the indicated hours **(B)**. RT-PCR was performed to determine the expression of human NEAT1 (NEAT1 and NEAT1_2) **(A, B)**. Shown are representative results from three independent experiments. **(C)** Quantitative real-time PCR was employed to detect the RNA levels of human NEAT1 (NEAT1 and NEAT1_2) in A549 cells infected with or without CA04 (MOI = 1) and H3N2 (MOI = 1). Data are represented as mean ± SD from three independent experiments. **(D, E)** Human cell lines, including 293T, A549, HeLa, and K562, were infected with or without WSN (MOI = 1) for 16 h. Expression of human NEAT1 (NEAT1 and NEAT1_2) in these cell lines was assessed by quantitative real-time PCR **(D)** and RT-PCR **(E)**. Data are represented as mean ± SD from three independent experiments. **(F)** RT-PCR was performed to examine the expression of cytoplasmic or nuclear human NEAT1 (NEAT1 and NEAT1_2) in A549 cells. β-actin served as a cytoplasmic control, and U6 as a nuclear control. Shown are representative results from three independent experiments. **(G)** Quantitative real-time PCR was employed to detect the RNA levels of human NEAT1 (NEAT1 and NEAT1_2) in A549 cells or 293T cells infected with or without SeV (MOI = 1), MDRV (MOI = 1), and PRV (MOI = 1). Data are represented as mean ± SD from three independent experiments. **(H)** Mouse NIH/3T3 cells were infected with or without PR8 (MOI = 1) for indicated times. Expression of mouse NEAT1 (mNEAT1 and mNEAT1_2) was assessed by quantitative real-time PCR. Data are represented as mean ± SD from three independent experiments. **(I)** Quantitative real-time PCR was employed to detect the RNA levels of mouse NEAT1 (mNEAT1 and mNEAT1_2) in PR8 (MOI = 1)-infected or mock-infected L929 cells. Data are represented as mean ± SD from three independent experiments. **(J)** WT C57BL/6J mice (6 weeks) were intranasally inoculated with WSN (5×10^4^ PFU/mL) for indicated time point, lung tissues were collected and lysed, and quantitative real-time PCR was performed to analyze the levels of mouse NEAT1 (mNEAT1 and mNEAT1_2). Data are represented as mean ± SD from three independent experiments. See also **Supplementary Figure S2**.

Next, we explored whether expression of NEAT1 could be induced by a broad spectrum of viruses. To this end, specific primers were designed to distinguish NEAT1 two transcriptional variants (NEAT1_1 and NEAT1_2) (**Supplementary Figure S2A**). We observed that at an MOI of 1, cytopathic effects were relatively minor at 16 hpi, whereas at an MOI of 2, extensive cell death was found. Based on this observation, we chose the MOI of 1 in the following studies. NEAT1 was upregulated by IAV strain PR8 in a virus dose-dependent manner in A549 cells (**Figure 2A**) and 293T cells (**Supplementary Figure S2B**). A time-course study also showed that NEAT1 was induced by IAV infection, and its level reached the highest point at 12 h post-infection (**Figure 2B**). NEAT1 expression was also significantly increased in A549 cells infected with other IAV strains, such as CA04, H3N2 (**Figure 2C**, **Supplementary Figures S2C, D**). Furthermore, the levels of NEAT1 induced by IAV were examined in several human cell lines, including 293T, A549, HeLa, and K562. Consistently, increased expression of NEAT1 was detected in these human cells infected with WSN (**Figures 2D, E**). Moreover, we explored the cellular localization of NEAT1 in IAV-infected A549 cells, and found that larger fraction of NEAT1 was accumulated in the nucleus (**Figure 2F**). Additionally, infections with other RNA viruses, including SeV and MDRV, and DNA viruses such as PRV also caused a significant increase in NEAT1 expression (**Figure 2G**; **Supplementary Figure S2E**). We then examined the expression of mNEAT1 upon the viral infection *in vitro* and *in vivo*. Similarly, mNEAT1 levels were dramatically elevated in NIH/3T3 cells infected either with IAV PR8 (**Figure 2H**; **Supplementary Figure S2F**) or IAV WSN (**Supplementary Figures S2G**; **S2H**). L929 mouse cell line was further employed and IAV-induced expression of mNEAT1 was also observed in the cells (**Figure 2I**; **Supplementary Figure S2I**). *In vivo* experiments exhibited that expression of mNEAT1 was significantly increased in mouse lungs challenged with the IAV (**Figure 2J**; **Supplementary Figure S2J**). Together, these experiments demonstrate that NEAT1 expression can be induced by infections with several viruses.

### Expression of NEAT1 is regulated by MDA5- and TLR3-dependent innate immune signaling pathways

**Figure 3:**
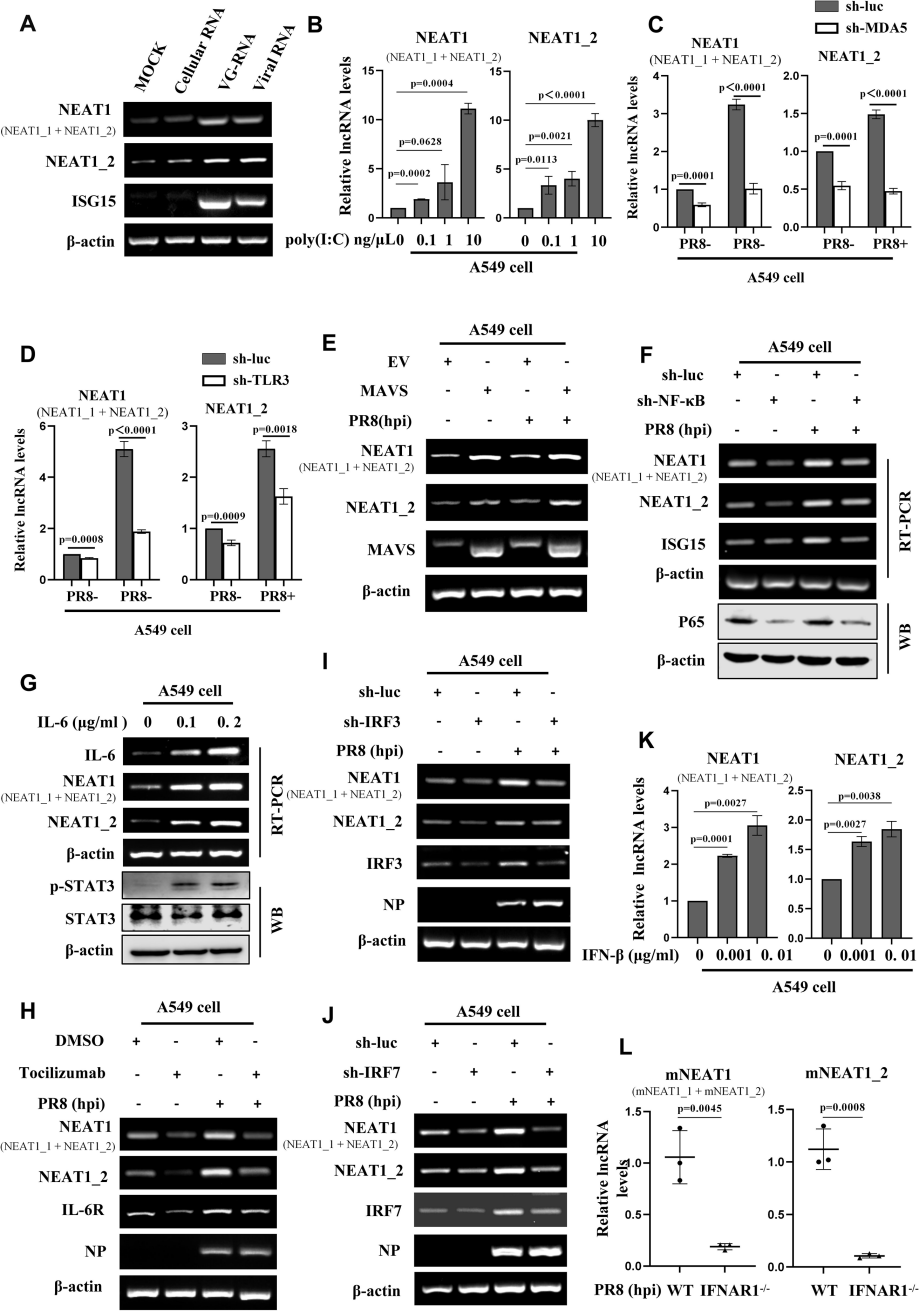
Expression of NEAT1 is regulated by MDA5- and TLR3-dependent innate immune signaling pathways. **(A)** The uninfected Cellular RNA, genomic RNA directly isolated from IAV (VG-RNA), and total RNA derived from PR8 (MOI = 1)-infected A549 cells (viral RNA) were transfected into A549 cells respectively, with a blank control group set as a reference. Detect the expression of human NEAT1 (NEAT1 and NEAT1_2) at 12 hours post-transfection by RT-PCR. Shown are representative results from three independent experiments. **(B)** A549 cells were transfected with poly(I:C) at the indicated concentrations for 4 h. The levels of human NEAT1 (NEAT1 and NEAT1_2) were examined by quantitative real-time PCR. Data are represented as mean ± SD from three independent experiments. **(C, D)** Quantitative real-time PCR was applied to examine the expression of human NEAT1 (NEAT1 and NEAT1_2) in MDA5 **(C)**- or TLR3 **(D)**-knockdown A549 cells infected with or without PR8 (MOI = 1) for 16 **(h)** Data are represented as mean ± SD from three independent experiments. **(E)** A549 cells overexpressing MAVS or empty vector (EV) were infected with PR8 or without (MOI = 1) for 16 **(h)** The expression of human NEAT1 (NEAT1 and NEAT1_2) was detected by RT-PCR. Shown are representative results from three independent experiments. **(F)** RT-PCR was applied to examine the expression of human NEAT1 (NEAT1 and NEAT1_2) in NF-κB-knockdown A549 cells infected with or without PR8 (MOI = 1) for 16 h. Shown are representative results from three independent experiments. **(G)** RT-PCR was employed to detect the RNA levels of human NEAT1 (NEAT1 and NEAT1_2) in IL-6-stimulated A549 cells. Shown are representative results from three independent experiments. **(H)** A549 cells were pretreated with the IL-6 receptor (IL-6R) inhibitor tocilizumab or DMSO for 2 h, followed by infection with or without PR8 (MOI = 1) for 16 **(h)** RT-PCR was used to detect the levels of human NEAT1 (NEAT1 and NEAT1_2). Shown are representative results from three independent experiments. **(I, J)** RT-PCR was performed to detect the expression of human NEAT1 (NEAT1 and NEAT1_2) in PR8 (MOI = 1)-infected or mock-infected IRF3- or IRF7-knockdown A549 cells. Shown are representative results from three independent experiments. **(K)** A549 cells were treated with IFN-β at indicated concentrations for 2 h, and expression of human NEAT1 (NEAT1 and NEAT1_2) was examined by quantitative real-time PCR. Data are represented as mean ± SD from three independent experiments. **(L)** IFNAR1 knockout (IFNAR1^-/-^) mice on C57BL/6J background or C57BL/6J WT mice (6 weeks) were infected with PR8 (5×10^4^ PFU/mL) for 24 h mNEAT1 levels were detected by quantitative real-time PCR. Data are represented as mean ± SD from three independent experiments. See also **Supplementary Figure S3**.

Since viral infection induced NEAT1 expression immediately within a few hours post-infection, we speculated that innate immune signaling pathway may regulate the expression of NEAT1 during viral infection. To test this possibility, A549 cells were treated with genomic RNA (VG-RNA) directly isolated from IAV, total RNA derived from IAV infected (viral RNA) or uninfected (cellular RNA) cells. The results showed that VG-RNA and viral RNA isolated from IAV-infected cells but not cellular RNA was able to upregulate the expression of NEAT1 (**Figure 3A**). We further tested whether double-stranded RNA (dsRNA), a common viral nucleic acid mimic, could regulate NEAT1 expression. To this end, we treated A549 cells with poly(I:C), a synthetic analog of dsRNA. Indeed, we found that poly(I:C) significantly induced a concentration-dependent upregulation of NEAT1 (**Figure 3B**; **Supplementary Figure S3A**).

RIG-I, MDA5, and TLR3 are pivotal pattern recognition receptors (PRRs) that detect the influenza virus infection (45). Upon sensing the influenza virus, these receptors become activated and engage with adaptor proteins to activate innate immune signaling. To address the role of these PRRs in induction of NEAT1 by IAV, A549 stable cell lines with RIG-I, MDA5, or TLR3 knockdown were generated. Interestingly, silencing MDA5 and TLR3, but not RIG-I led to a decrease in NEAT1 expression (**Figures 3C, D**; **Supplementary Figures S3B-S3D**). Overexpression of MAVS caused an obvious upregulation of NEAT1 expression during IAV infection (**Figure 3E**).

It is well known that NF-κB/IL-6 pathway is a main inducer of STAT3. Thus, we determined whether the upregulation of NEAT1 by viral infection was associated with NF-κB/IL-6 signaling. Indeed, we observed that knocking down transcription factor NF-κB with specific sh-RNA had significant effect on the NEAT1 levels in cells infected with or without IAV (**Figure 3F**). As expected, NEAT1 expression in IL-6 treated A549 cells was elevated in a dose-dependent manner (**Figure 3G**). Treatment with tocilizumab, an irreversible inhibitor of IL-6 receptor (IL-6R), significantly decreased the IAV-induced NEAT1 production compared with the control (**Figure 3H**).

Next, effects of silencing IRF3 or IRF7 on NEAT1 expression was examined. Knockdown of these transcription factors also impaired the IAV-induced expression of NEAT1 as compared with the controls (**Figures 3I, J**). Moreover, IFN-β stimulation resulted in a concentration-dependent upregulation of NEAT1 expression (**Figure 3K**; **Supplementary Figure S3E**). Consistently, IFNAR1 deficiency in mice (IFNAR1^-/-^) caused a decrease in the induction of mNEAT1 by IAV (**Figure 3L**). These data indicate that IAV-induced NEAT1 expression is regulated by MDA5- and TLR3-dependent innate immune signaling pathways involving NF-κB, IL-6 and IFN-β.

### Altering NEAT1 expression has significant effects on the viral replication *in vitro*

**Figure 4:**
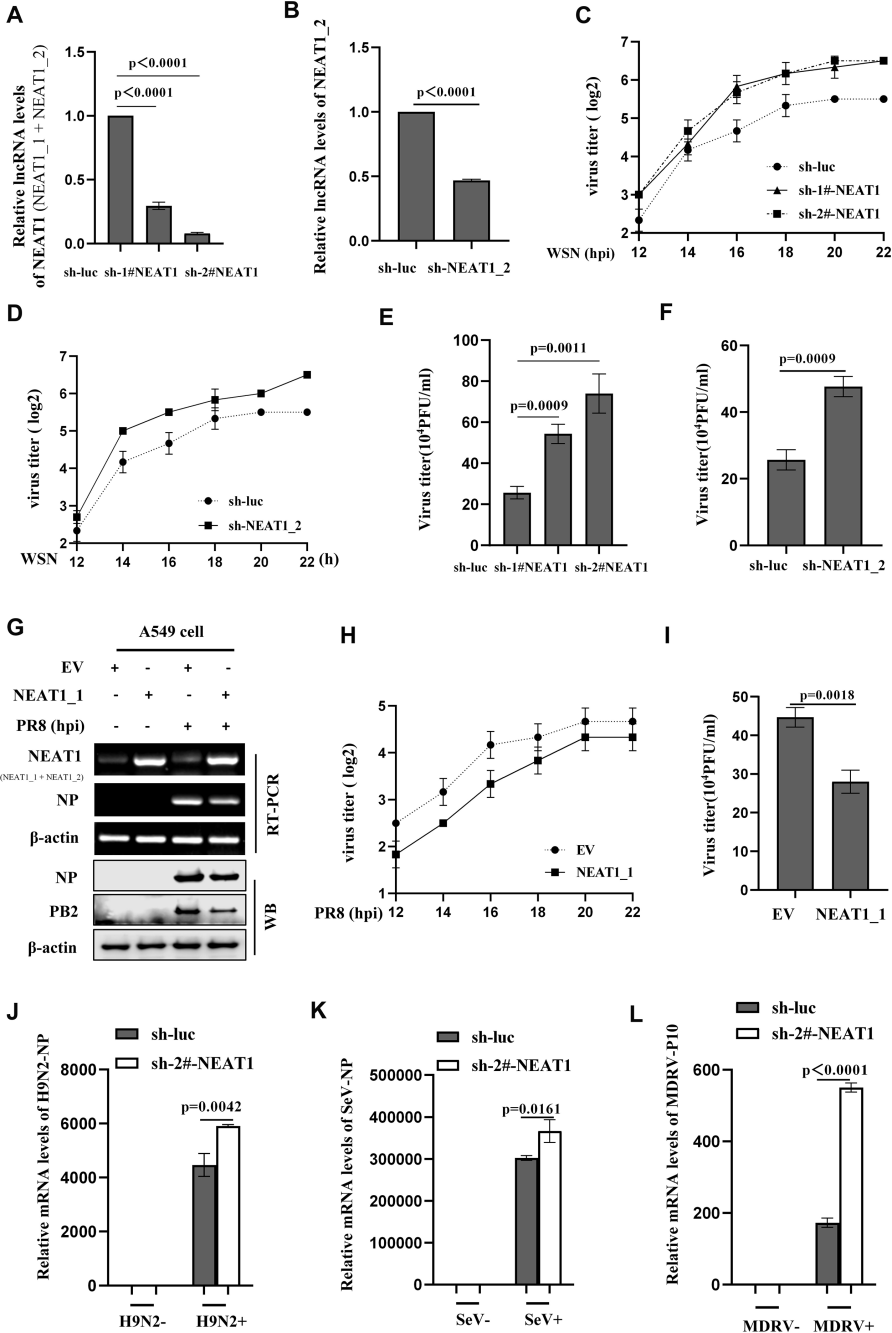
Altering NEAT1 expression has significant effects on the viral replication *in vitro.* **(A, B)** The knockdown efficiency of shRNAs specifically targeting NEAT1 (sh-1#-NEAT1, sh-2#-NEAT1, and sh-NEAT1_2) in A549 cells was determined by quantitative real-time PCR. Data are represented as mean ± SD from three independent experiments. **(C, D)** IAV titers in supernatants of WSN (MOI = 1)-infected NEAT1 (NEAT1 or NEAT1_2) -knockdown A549 cells were measured by hemagglutination assay. Data are represented as mean ± SD from three independent experiments. **(E, F)** Supernatants from NEAT1-knockdown and control A549 cells infected with IAV WSN strain were collected at 16 **(h)** IAV titers were examined by plaque-forming assay. Data are represented as mean ± SD from three independent experiments. **(G, H)** The levels of NEAT1 in PR8 (MOI = 1)-infected or mock-infected NEAT1_1-overexpressing A549 cells and control cells were measured by RT-PCR **(G)**. Viral titers in supernatants were measured by hemagglutination assay **(H)**. Data are represented as mean ± SD from three independent experiments. **(I)** Plaque-forming assay was employed to detect the viral titers in supernatants of PR8 (MOI = 1)-infected NEAT1_1-overexpressing and control A549 cells. Shown are representative results from three independent experiments. **(J)** The mRNA levels of H9N2 NP in NEAT1-knockdown and luciferase control A549 cells were measured by quantitative real-time PCR. Data are represented as mean ± SD from three independent experiments. **(K, L)** The mRNA levels of SeV NP **(K)** or MDRV P10 **(L)** in NEAT1-knockdown and luciferase control 293T cells were measured by quantitative real-time PCR. Data are represented as mean ± SD from three independent experiments. See also **Supplementary Figure S4**.

To evaluate the role of NEAT1 in IAV infection, we examined the effects of altering NEAT1 expression on the viral replication and host antiviral responses. For this, we designed three short hairpin RNAs (shRNAs) to generate NEAT1 knockdown A549 cell lines (**Supplementary Figure S4A**). The interference efficiency of sh-2#-NEAT1 was superior to sh-1#-NEAT1 for disruption of total NEAT1 expression (NEAT1_1 and NEAT1_2 transcripts), and sh-NEAT1_2 specifically knocked down the expression of NEAT1_2 transcript (**Figures 4A, B**; **Supplementary Figures S4B-S4D**).

Subsequently, we employed the aforementioned shRNAs to investigate the role of NEAT1 in IAV replication. Results showed that silencing NEAT1 (NEAT1_1 and NEAT1_2) caused a significant increase in mRNA levels of viral NP and NS1 in the IAV-infected cells (**Supplementary Figures S4E**; **S4F**). Furthermore, we analyzed viral load by HA assay, and data displayed that silencing NEAT1 markedly promoted the WSN replication (**Figures 4C, D**). Similarly, the results from PFA assay also revealed that knocking down NEAT1 led to a significant increase in the virus titers (**Figures 4E, F**).

On the other hand, we determined the effects of NEAT1 overexpression on IAV replication. Since NEAT1_2 transcript has a large size (21-23Kb), we chose NEAT1_1 for this study and generated stable A549 cells overexpressing NEAT1_1 (**Figure 4G** and **Supplementary Figure S4G**). Experiments using RT-PCR, quantitative real-time PCR, and Western blotting consistently showed the decreased mRNA and protein levels of viral NP in NEAT1_1 overexpressing A549 cells compared to those in control cells upon IAV infection (**Figure 4G**; **Supplementary Figure S4H**). Both PFA and HA assay exhibited that virus titers were remarkably downregulated in A549 cells with NEAT1_1 overexpression (**Figures 4H, I**). In addition, we investigated the effects of NEAT1 on infections with H9N2 strain of avian influenza virus (AIV) and other RNA viruses, such as MDRV and SeV. Similarly, silencing NEAT1 resulted in an increase in the mRNA levels of H9N2 NP, SeV NP, and MDRV P10 (**Figures 4J–L**). These findings suggest that lncRNA NEAT1 may inhibit the replication of multiple RNA viruses.

### NEAT1 knockout mice are more susceptible to IAV infection

To uncover the regulatory role of NEAT1 in IAV infection, we wished to establish a more physiological model system. To this end, we employed the mNEAT1 knockout (NEAT1^-/-^) mice generated by CRISPR/Cas9-based genome editing (**Supplementary Figure S5A**). RT-PCR and quantitative real-time PCR assays revealed that expression of mNEAT1 was completely lost in heart, liver, spleen, lung, kidney, and brain of homozygote NEAT1 knockout mice (**Supplementary Figures S5B**; **S5C**).

**Figure 5:**
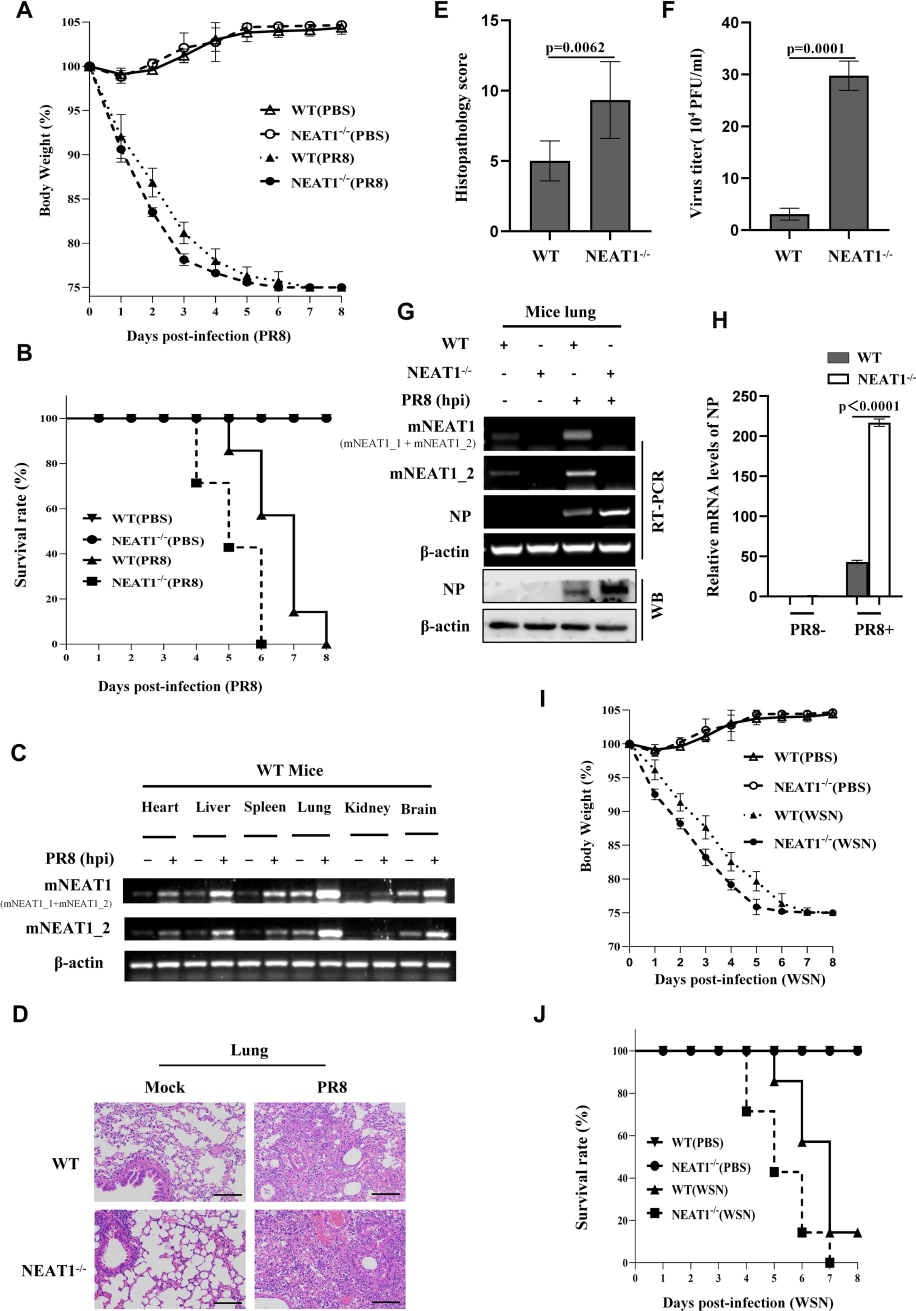
NEAT1 knockout mice are more susceptible to IAV infection. **(A, B)**, and **(D-H)** C57BL/6J WT mice and NEAT1^-/-^ mice (6 weeks) were intranasally inoculated with PR8 (5×10^4^ PFU/mL). The body weight change **(A)** and survival rate **(B)** of mice were monitored (6 mice for each Mock group and 10 mice for each PR8 infection group). Mice were intranasally inoculated with PR8 for 48 h. Shown are representative micrographs of lung sections of the indicated mice stained with hematoxylin and eosin (H and E) **(D)**. Scale bars, 100 µm. Shown are lung pathology score **(E)**. The viral titers in the lungs from WT and NEAT1^-/-^ mice at 2 dpi were determined by plaque-forming assay **(F)**. Data are represented as mean ± SD from three independent experiments. The levels of NP from mice lungs at 2 dpi were measured by RT-PCR **(G)**, Western blotting **(G)**, and quantitative real-time PCR **(H)**. **(C)** Expression levels of mNEAT1 in different organs (heart, liver, spleen, lung, kidney, and brain) of WT mice infected with or without PR8 were examined by RT-PCR. Shown are representative results from three independent experiments. **(I, J)** Shown are the body weight change **(I)** and survival rates **(J)** of C57BL/6J WT mice and NEAT1^-/-^ mice (6 weeks) intranasally inoculated with WSN (5×10^4^ PFU/mL) (6 mice for each Mock group and 10 mice for each WSN infection group). Body weight was measured every day. See also **Supplementary Figure S5**.

The NEAT1^-/-^ and WT mice were then intranasally inoculated with IAV PR8 virus, and the influence of NEAT1 knockout on IAV virulence and infection kinetics was examined. Indeed, body-weight loss of NEAT1^-/-^ mice was clearly higher than that observed in WT groups infected with the IAV (**Figure 5A**). Under our experimental condition, all NEAT1^-/-^ mice died within 6 days post infection (dpi), whereas approximately 60% of WT mice still survived at this time point (**Figure 5B**). The NEAT1^-/-^ mice displayed the faster body temperature drops than that of WT animals after challenge with IAV (**Supplementary Figures S5D**). There were varying degrees of elevation in mNEAT1 levels in heart, liver, spleen, lung, kidney, and brain of WT mice upon the IAV infection (**Figure 5C**). Notably, NEAT1^-/-^ mice exhibited a significantly greater extent of acute lung injury compared to the WT mice (**Supplementary Figure S5E**). Consistently, pathologic examination by hematoxylin and eosin (H&E) staining displayed more severe edema and increased infiltration of inflammatory cells in the lungs of NEAT1^-/-^ mice than those observed in the WT controls (**Figures 5D, E**). Moreover, IAV titers were remarkably increased in lung tissues derived from NEAT1^-/-^ mice compared with those in WT mice (**Figure 5F**). Viral NP mRNA and protein in lungs of NEAT1^-/-^ mice were markedly increased as compared with those in the WT groups (**Figures 5G, H**). Additionally, NEAT1 knockout mice were infected with the WSN strain of IAV to evaluate the effect of NEAT1 deficiency on WSN replication. A time-course analysis demonstrated that NEAT1 knockout mice exhibited a faster rate of body weight loss and a reduced survival rate than control mice challenged with IAV WSN strain (**Figures 5I, J**). Together, these results reveal that NEAT1 deficiency renders mice more susceptible to IAV infection.

### Activated STAT3 suppresses IAV replication through positively regulating the expression of NEAT1

**Figure 6:**
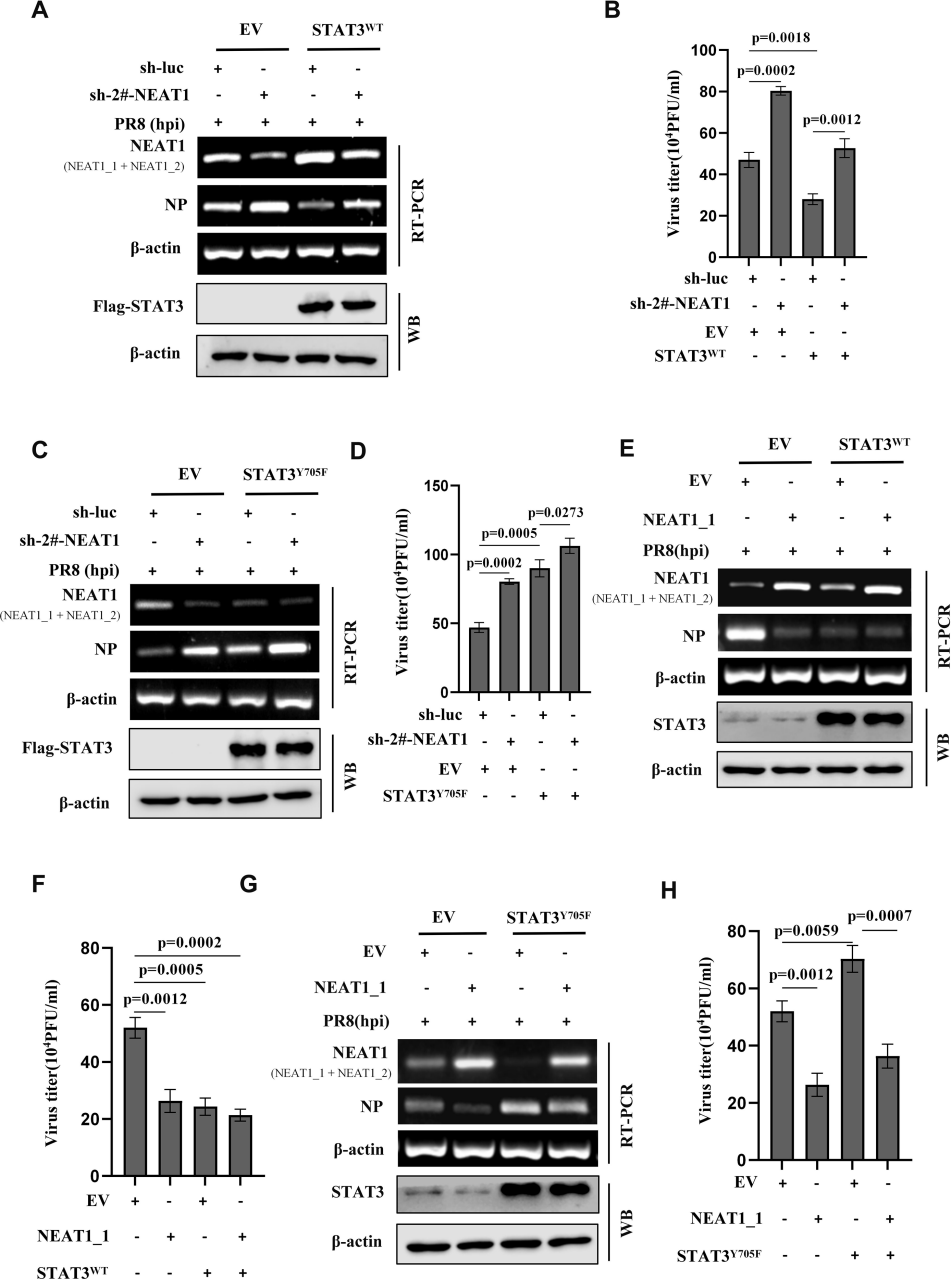
Activated STAT3 suppresses IAV replication through positively regulating the expression of NEAT1. **(A-D)** A549 cell lines stably expressing Flag-STAT3^WT^ **(A, B)**, Flag-STAT3^Y705F^ **(C, D)**, or empty vector (EV) were transfected with sh-luc or sh-2#-NEAT1, followed by infection with PR8 virus (MOI = 1) for 16 h. The mRNA levels of viral NP in the cells were examined by RT-PCR **(A, C)**, and the viral titers in the supernatants of these cells were examined by plaque-forming assay **(B, D)**. Data are represented as mean ± SD from three independent experiments. **(E, F)** Flag-STAT3^WT^ overexpressing A549 cells and control cells were transfected with EV or NEAT1_1, followed by infection with PR8 virus (MOI = 1) for 16 h. The mRNA levels of viral NP were examined by RT-PCR **(E)**, and the viral titers in the supernatants of these cells were examined by plaque-forming assay **(F)**. Data are represented as mean ± SD from three independent experiments. **(G, H)** Flag-STAT3^Y705F^ overexpressing A549 cells and control cells were transfected with EV or NEAT1_1, followed by infection with PR8 virus (MOI = 1) for 16 h. The mRNA levels of viral NP in the cells were examined by RT-PCR **(G)**, and the viral titers in the supernatants of these cells were examined by plaque-forming assay **(H)**. Data are represented as mean ± SD from three independent experiments. See also **Supplementary Figure S6**.

Since our previous research has shown that activated STAT3 can effectively inhibit the IAV infection and above results reveal that IAV-induced NEAT1 expression is regulated by the STAT3, we determined whether NEAT1 might serve as a regulatory RNA in the STAT3-mediated antiviral immunity. To address this, A549 cell lines stably overexpressing STAT3^WT^ was generated. We observed that viral NP expression exhibited a clear reduction in STAT3-overexpressing cells challenged with IAV PR8 (**Figure 6A**). However, shRNA-based NEAT1 knockdown in the STAT3^WT^-overexpressing cells restored the mRNA level of NP and viral titers to comparable levels with those of the control cells infected with IAV (**Figures 6A, B**). In addition, silencing NEAT1 expression in cells overexpressing STAT3^Y705F^ also caused a upregulation of NP expression and an increased viral titer compared to the control STAT3^Y705F^ cells group (**Figures 6C, D**). These data suggest a profound effect of NEAT1 on the STAT3-mediated immunity against IAV infection.

To further explore the implication of NEAT1 in IAV infection, we constructed the NEAT1_1 overexpression vectors. When STAT3^WT^-overexpressing cells were transfected with the NEAT1_1 in a lentiviral vector, a reduction in viral NP and viral titers was observed, as compared with the control cells (**Figures 6E, F**). Importantly, following the transfection of NEAT1_1-overexpressing plasmids into STAT3^Y705F^ A549 cells and subsequent infection with IAV, the mRNA level of viral NP was downregulated compared to that in control STAT3^Y705F^ A549 cells (**Figure 6G**). This finding was further confirmed by PFA assay, demonstrating that the overexpression of NEAT1_1 in STAT3^Y705F^ A549 cells resulted in a significant reduction in viral titers (**Figure 6H**). In addition, we used an unrelated lncRNA-up4 as a control. LncRNA-up4 (TCONS_00098959) is a chicken lncRNA identified by Chen’s laboratory (**Supplementary Figure S6A**). The data showed that overexpression of the chicken lncRNA in STAT3^Y705F^ A549 cells had no significant effect on mRNA levels of viral NP compared with the control (**Supplementary Figure S6B**). Collectively, the results imply that activated STAT3 suppresses IAV replication probably through positively regulating the expression of NEAT1.

### NEAT1 is involved in regulation of innate antiviral responses

**Figure 7:**
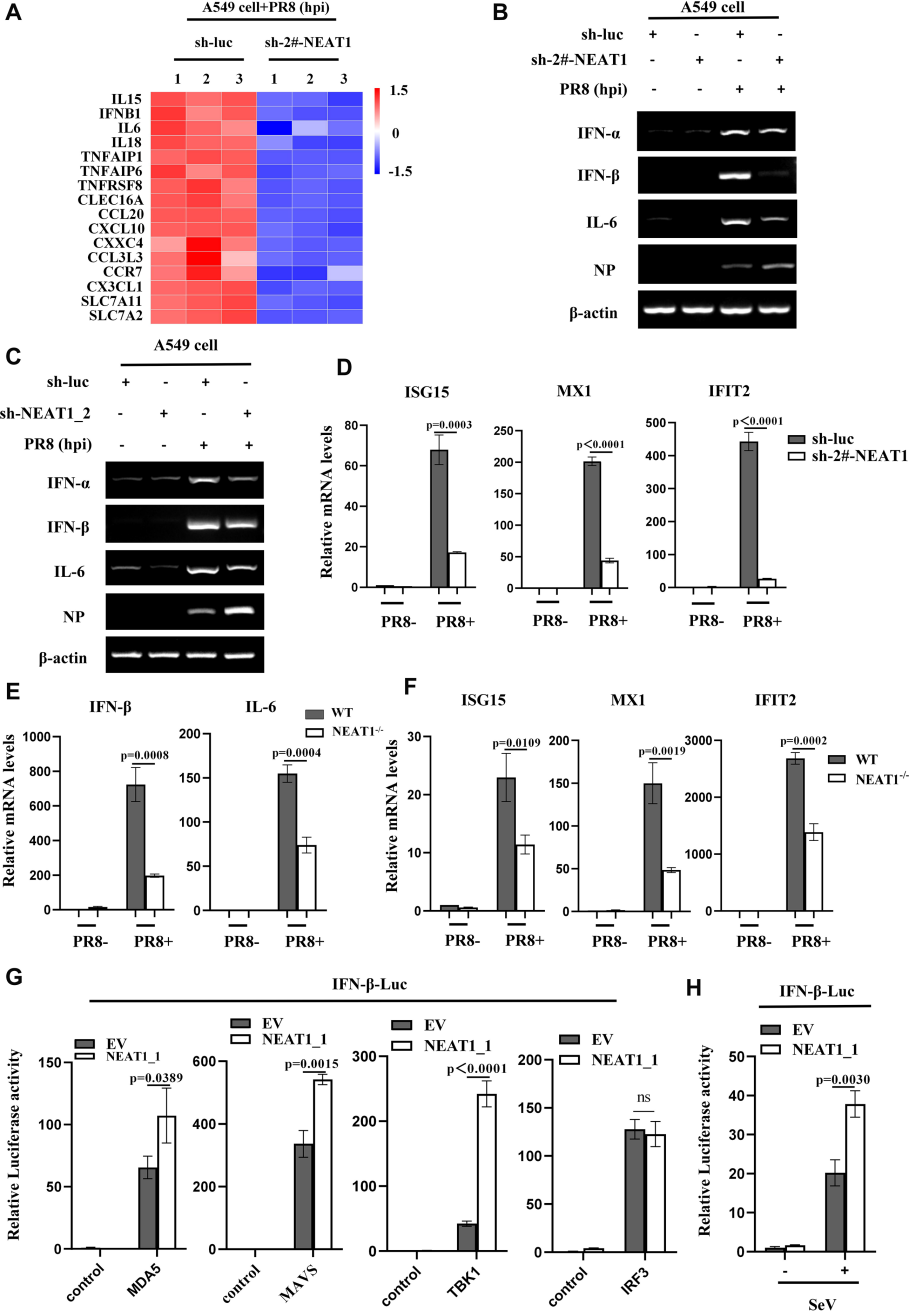
NEAT1 is involved in regulation of innate antiviral responses. **(A)** RNA-Seq analysis was conducted on control and NEAT1-knockdown A549 cells infected with PR8 (MOI = 1) for 16 h (fold change >2, *P* < 0.05).**(B-D)** Control and (2#-NEAT1 or NEAT1_2)-knockdown A549 cells were infected with or without PR8 (MOI = 1) for 16 h. The mRNA levels of IFN-a, IFN-β, or IL-6 were examined by RT-PCR **(B, C)**, and the mRNA levels of ISG15, MX1, and IFIT2 were examined by quantitative real-time PCR **(D)**. Data are represented as mean ± SD from three independent experiments. **(E, F)** Quantitative real-time PCR was performed to analyze the levels of IFN-β **(E)**, IL-6 **(E)**, ISG15 **(F)**, MX1 **(F)**, and IFIT2 **(F)** in the lungs from C57BL/6J WT mice and NEAT1^-/-^ mice (6 weeks) at 48 h post PR8 virus (5×10^4^ PFU/mL) infection. Data are represented as mean ± SD from three independent experiments. **(G)** Control and NEAT1_1 overexpressing 293T cells were transfected with IFN-β luciferase reporter and indicated plasmids. 24 hours post-transfection, the cells were harvested for luciferase assay. Data are represented as mean ± SD from three independent experiments. **(H)** Control and NEAT1_1 overexpressing 293T cells were transfected with IFN-β luciferase reporter and pRL-TK plasmids. After 24 hours, the cells were infected with or without SeV (MOI = 1). After 16 hours, the cells were collected for luciferase assay. Data are represented as mean ± SD from three independent experiments. See also **Supplementary Figure S7**.

Next, we sought to dissect how NEAT1 suppresses the viral replication. To this end, we performed RNA-Seq to analyze differentially expressed mRNAs between NEAT1 knockdown and control A549 cells infected with PR8 (GEO: GSE306614) (**Supplementary Figure S7A**). Notably, our RNA-Seq analysis displayed that expression levels of type I IFNs and some antiviral molecules, which play roles in restricting viral infections, were significantly reduced in NEAT1 knockdown A549 cells as compared to the control cells upon IAV infection (**Figures 7A, B**). Consistently, NEAT1_2 knockdown also significantly decreased the mRNA levels of type I IFNs and IL-6 in cells infected with PR8 (**Figure 7C**).

The results from RNA-Seq were also confirmed by examining the expression of several ISGs in NEAT1 knockdown cells. The data showed that disruption of NEAT1 led to a clear reduction in the mRNA levels of ISG15, MX1, and IFIT2 as compared with the control (**Figure 7D**, **Supplementary Figures S7B**; **S7C**). Moreover, NEAT1^-/-^ mice displayed a significant decrease in mRNA levels of IFN-β, IL-6 and ISGs compared to those in WT mice challenged with IAV (**Figures 7E, F**; **Supplementary Figures S7D, S7E**), indicating that NEAT1 deficiency may suppress the antiviral innate immunity. Together, these results suggest that NEAT1 functions in restricting viral replication, likely through upregulation of innate immune responses.

To decode the potential mechanism by which NEAT1 promotes IFN response during IAV infection, we determined whether NEAT1 facilitated RLR-dependent signaling. Thus, IFN-β luciferase reporter system was employed, and NEAT1_1 overexpressing cells and controls were transfected with the reporter vector and either MDA5, MAVS, TBK1, or IRF3 expression plasmid. The data showed that NEAT1_1 significantly promoted MDA5-, MAVS, and TBK1-mediated IFN-β luciferase activity but not IRF3-induced IFN-β luciferase activity, suggesting that NEAT1 might promote innate immune signaling by regulating TBK1 activation (**Figure 7G**). In addition, the NEAT1_1 overexpressing cells were transfected with the IFN-β luciferase reporter and infected with SeV. As expected, the overexpression of NEAT1_1 significantly augmented the SeV-induced IFN-β luciferase activity (**Figure 7H**). Furthermore, RNA immunoprecipitation (RIP) assay was performed, which exhibited no direct association between TBK1 and NEAT1 in cells infected with IAV (**Supplementary Figures S7F**, **S7G**), suggesting that NEAT1 may modulate the TBK1 activation through some intermediate molecule(s). Identification of these molecule(s) warrants further investigation in the future.

## Discussion

STAT3 protein has various biological activities, especially plays an important role in antiviral immunity (46, 47). LncRNA is widely involved in the pathogenic process of viruses (48). In recent years, studies have shown that STAT3 interact with lncRNA, such as FGD5-AS1, LINC00908, and FOXD2-AS1 to regulate the occurrence and development of cancer (49, 50). However, the STAT3-lncRNA interaction and its role in antiviral immune response are still unclear. The experiments shown in this study demonstrate that activated-STAT3 significantly promotes the expression of lncRNA NEAT1 during IAV infection, and NEAT1 functions downstream of STAT3 signaling to enhance antiviral responses and thereby suppress viral replication.

Although much emphasis has been placed on investigating host lncRNAs as important regulators in different biological settings, few lncRNAs have been implicated in antiviral immunity. At present, only a small part of the research on the relationship between lncRNAs associated with IAV pathogenesis (51). The expression of the lncRNAs, such as NRAV (52), TSPOAP1-AS1, and IFITM4P are induced by IAV infection and plays various roles in IAV infection (53). Here, using the STAT3^Y705F/+^ mice model, we conducted transcriptome analysis of lncRNAs in animals infected with or without IAV. The data provide evidence that disruption of STAT3 Y705 phosphorylation significantly inhibited the expression of the lncRNA NEAT1 induced by IAV infection. Notably, *in vivo* experiments showed that altering the expression of STAT3 or its activation significantly affected the production of NEAT1 during IAV infection. For example, silencing STAT3 significantly attenuated the NEAT1 RNA levels. Disruption of STAT3 Y705 phosphorylation suppressed the expression of NEAT1. Moreover, increased expression of NEAT1 could be detected in IAV-infected A549 cell lines stably expressing either wild-type STAT3 (STAT3^WT^), or constitutively active mutant of STAT3 (STAT3^D661V^). The results obtained from a series of experiments reveal that expression of NEAT1 was regulated via MDA5 and TLR3 signaling pathways involving NF-κB, IL-6 and IFN-β during IAV infection. This finding is consistent with a previous report that depletion of MDA5 and TLR3, but not RIG-I, affected poly (I:C)-induced NEAT1 expression (54). Our previous research has shown that STAT3 markedly suppressed the replication of IAV (19). Therefore, together these observations, we hypothesized that IAV-induced NEAT1 expression, regulated through STAT3 signaling activated by the PRRs- and NF-κB-dependent pathways, may be implicated in STAT3-mediated antiviral innate immunity.

Consequently, we investigated the regulatory role of NEAT1 in the STAT3-mediated antiviral immune response. NEAT1 has two transcriptional variants, NEAT1_1 and NEAT1_2, which have been found to be expressed in various human and mice cells. Experiments demonstrate that both NEAT1_1 and NEAT1_2 were significantly upregulated during the IAV infection and infections with ssRNA virus (SeV), dsRNA virus (MDRV), and DNA virus (PRV). Strikingly, altered expression of NEAT1 had a profound effect on replication of some viruses, such as IAV, SeV, and MDRV. For instance, diminished expression of NEAT1 significantly promoted the multiplication of IAV in A549 cells. Silencing NEAT1 significantly enhanced the replication of SeV and MDRV. In contrast, overexpression of NEAT1_1 significantly inhibited IAV replication in host cells. These observations were consistent with previous studies showing that infection with Hantaan virus (HTNV) induces NEAT1 expression that promotes robust IFN responses and thereby inhibits viral replication (55). Our previous experiments demonstrated that IAV production was significantly increased in STAT3^Y705F^ host (19). Importantly, here we found that levels of viral NP and viral titers were markedly reduced following overexpression of NEAT1_1 in A549 cells expressing STAT3^Y705F^, as compared to control cells. This finding was consistent with a study claiming that herpes simplex virus-1 (HSV-1) infection upregulates NEAT1 expression in a STAT3-dependent manner (44).

To study the function of NEAT1 in the physiological state, we employed NEAT1^-/-^ mice. The *in vivo* studies showed that NEAT1^-/-^ mice exhibited more susceptible to IAV infection, as evidenced by increased lung viral replication, aggravated acute lung injury, accelerated weight loss, and reduced survival rates. However, the *in vivo* interaction between NEAT1 and STAT3, as well as the precise mechanism underlying the antiviral effects of NEAT1 *in vivo*, requires further investigation. In addition, the fact that NEAT1 can be induced by several viruses suggest that it may be more broadly involved in many viral pathogenesis. However, this remains to be further determined *in vivo*.

Furthermore, we performed an in-depth investigation into the regulatory function of NEAT1 in innate immunity. Our data reveal that NEAT1 is involved in regulating innate antiviral responses through enhancing the production of several antiviral molecules including IFNs, ISG15, MX1, and IFIT2. Moreover, the finding suggests that NEAT1 may contribute to TBK1 activation, thereby enhancing innate immune response mediated by STAT3. Taken together, these findings indicate that NEAT1 may function downstream of STAT3 and act as a regulatory RNA to modulate the host innate immunity against IAV infection. The precise mechanism needs to be further investigated.

Given that a dose-dependent upregulation of NEAT1 occurs during IAV infection, the detection of differentially expressed NEAT1 in the serum of influenza patients may represent a potential diagnostic approach for monitoring disease progression. Some studies have indicated that NEAT1 may function as a potential therapeutic target for viral infection. For instance, Pandey et al. showed that reduced expression of NEAT1 is associated with the development of severe dengue phenotypes during dengue virus infection (56). Another study demonstrates that NEAT1 is linked to the onset of cytokine storms in SARS-CoV-2 infection (57). Together, these data indicate that NEAT1 could be a promising therapeutic target for treating viral diseases.

In summary, we have elucidated a mechanism by which STAT3 exerts a significant influence on antiviral immunity through the regulation of NEAT1 expression (**Supplementary Figure S8**). This study also furnishes evidence supporting the involvement of lncRNAs in STAT3-regulated antiviral immunity. Further research should focus on identifying the interplay between more lncRNAs and STATs, as well as characterizing the precise mechanisms underlying their interactions.

## Conclusion

In this study, we establish that IAV-induced expression of lncRNA NEAT1 is regulated by activated STAT3 *in vitro* and *in vivo*, which contributes to STAT3-mediated antiviral immunity. NEAT1 functions downstream of STAT3 signaling to impair viral replication by upregulating type I IFNs-mediated antiviral responses. Moreover, deficiency of NEAT1 in mice significantly enhances the IAV replication and virulence in the animals. These findings provide valuable insights into the interaction between virus and host immune system, and contribute to establishment of scientific foundation for developing lncRNA-based antiviral strategies.

## Data Availability

The original contributions presented in the study are included in the article/supplementary files, further inquiries can be directed to the corresponding author/s.
