# Supplementary material for: Expression of NEAT1 induced by influenza virus infection is regulated by activated STAT3 and contributes to STAT3-mediated antiviral immunity
Source: Front Immunol. 2025 Oct 23;16:1693884. doi: 10.3389/fimmu.2025.1693884 (PMC12588940; doi:10.3389/fimmu.2025.1693884)
Supplement: Supplementary file 1 [file DataSheet1.pdf]

Supplemental Materials and Methods

RNA immunoprecipitation

RNA immunoprecipitation (RIP) was performed as follows: the indicated antibody-conjugated beads or control beads were incubated with total protein extracted from the cell lysates, and the beads were then used to precipitate the protein-RNA complex. Non-specifically bound proteins and RNAs were washed with high-salt buffer containing Tween-20, and the complexes were analyzed using RT-PCR and Western blotting.

Fig.S1

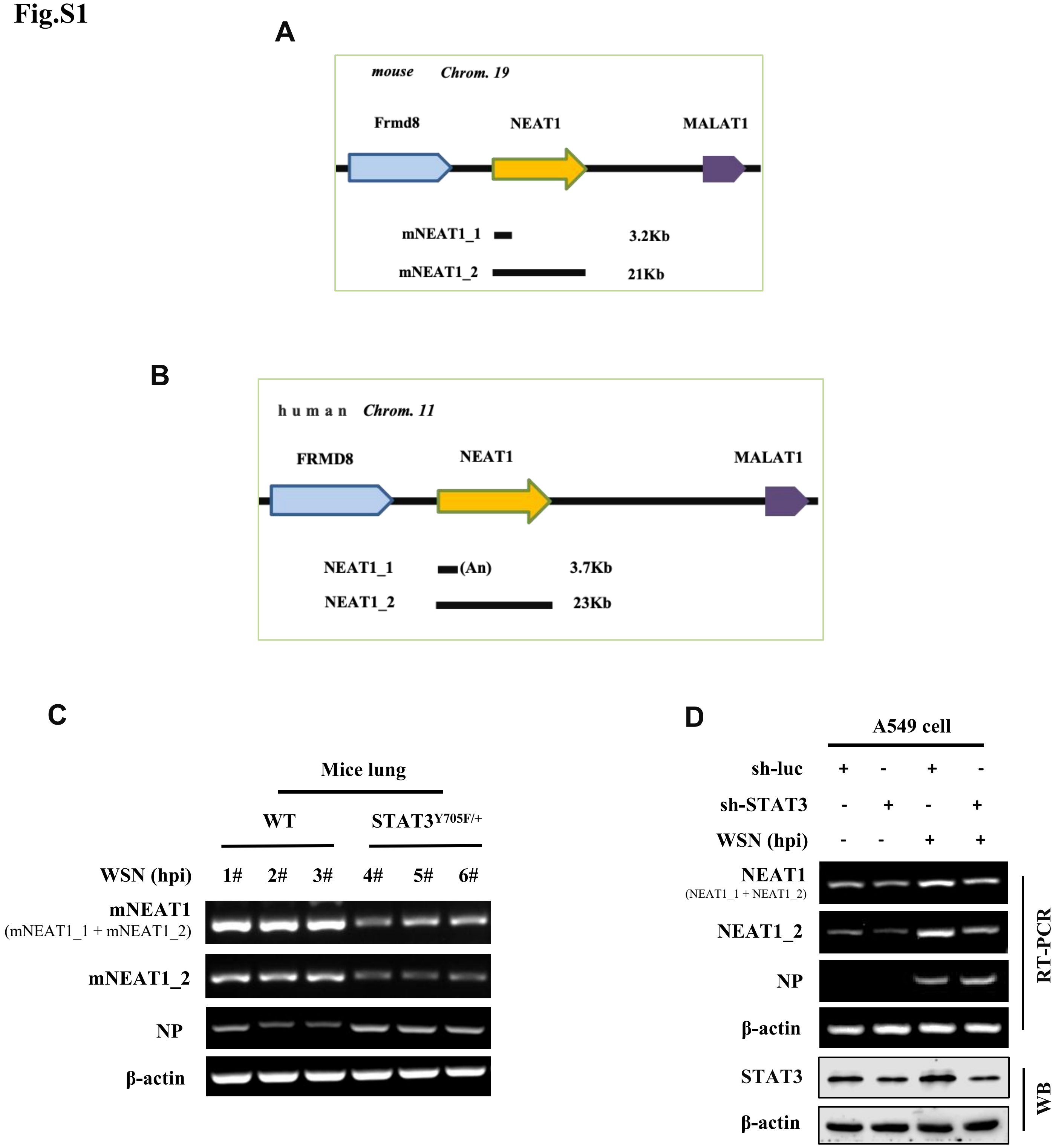
A
mouse Chrom. 19
Frmd8
NEAT1
MALAT1
mNEAT1_1
3.2Kb
mNEAT1_2
21Kb
B
human Chrom. 11
FRMD8
NEAT1
MALAT1
NEAT1_1
(An)
3.7Kb
NEAT1_2
23Kb
C
Mice lung
WT
STAT3Y705F/+
WSN (hpi)
1# 2# 3# 4# 5# 6#
mNEAT1
(mNEAT1_1 + mNEAT1_2)
mNEAT1_2
NP
β-actin
D
A549 cell
sh-luc + - + -
sh-STAT3 - + - +
WSN (hpi) - - + +
NEAT1
(NEAT1_1 + NEAT1_2)
NEAT1_2
NP
β-actin
STAT3
β-actin
RT-PCR
WB

Figure S1 Activated STAT3 is required for IAV-induced expression of NEAT1 *in vitro* and *in vivo*

(A and B) Shown is a paradigm of the genomic location of mouse NEAT1 (A) or human NEAT1 (B).

(C) 6 weeks-old BALB/c wild type (WT) mice or $STAT3^{Y705F/+}$ mice on BALB/c background were infected intranasally with WSN (5×10^4 PFU/mL) for 24 h. RT-PCR was performed to detect the expression of mouse NEAT1 (mNEAT1 and mNEAT1_2) . Shown are representative results from three independent experiments.

(D) A549 cells expressing specific shRNAs targeting STAT3 and luciferase (sh-luc), were infected with or without WSN (MOI=1) for 16 h. The expression of human NEAT1 (NEAT1 and NEAT1_2) were detected by RT-PCR. Shown are representative results from three independent experiments.

Fig.S2

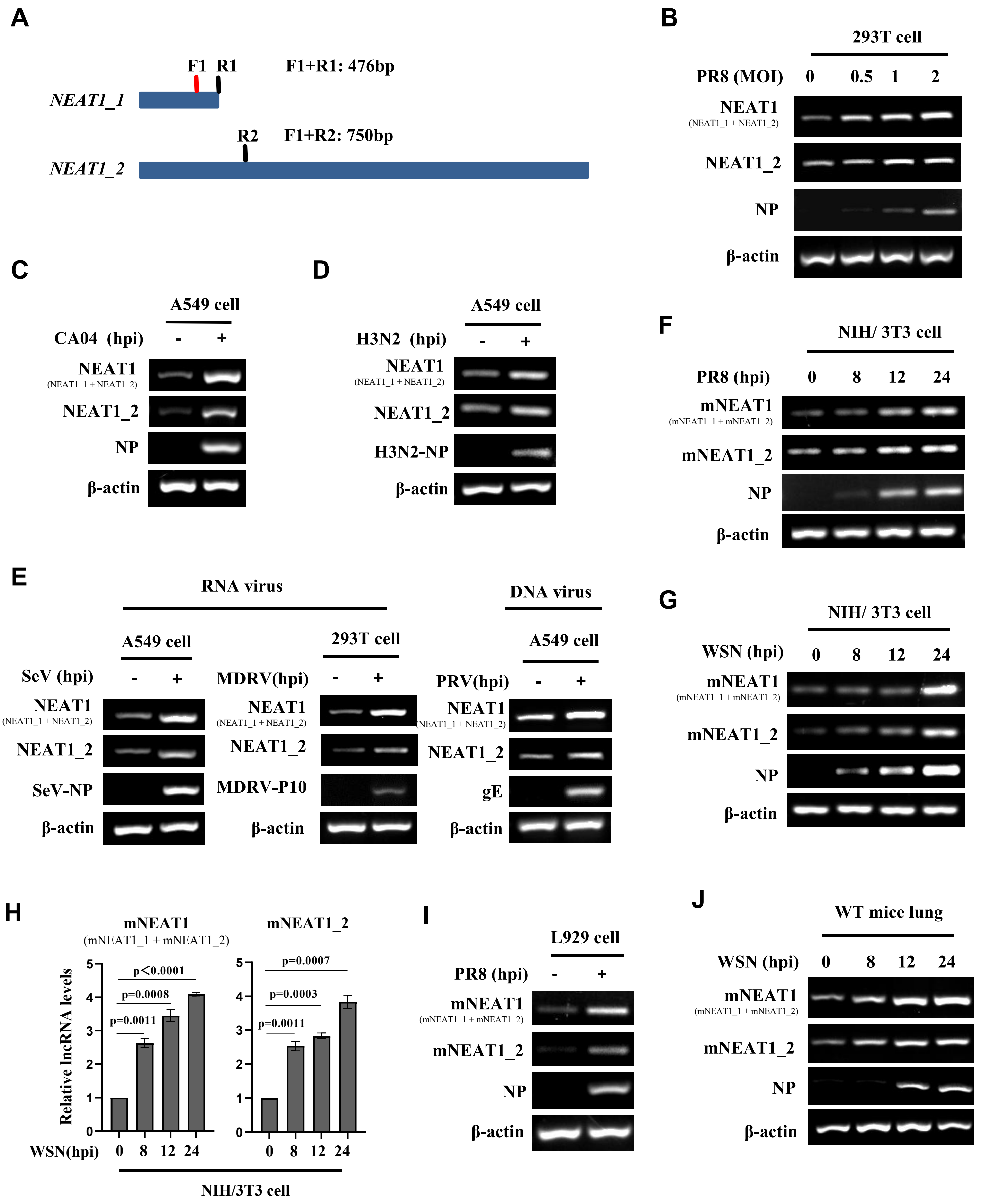

A
F1 R1
F1+R1: 476bp
NEAT1_1
R2
F1+R2: 750bp
NEAT1_2
B
293T cell
PR8 (MOI) 0 0.5 1 2
NEAT1 (NEAT1_1 + NEAT1_2)
NEAT1_2
NP
β-actin
C
A549 cell
CA04 (hpi) - +
NEAT1 (NEAT1_1 + NEAT1_2)
NEAT1_2
NP
β-actin
D
A549 cell
H3N2 (hpi) - +
NEAT1 (NEAT1_1 + NEAT1_2)
NEAT1_2
H3N2-NP
β-actin
F
NIH/ 3T3 cell
PR8 (hpi) 0 8 12 24
mNEAT1 (mNEAT1_1 + mNEAT1_2)
mNEAT1_2
NP
β-actin
E
RNA virus
DNA virus
A549 cell
293T cell
A549 cell
SeV (hpi) - +
MDRV(hpi) - +
PRV(hpi) - +
NEAT1 (NEAT1_1 + NEAT1_2)
NEAT1_2
SeV-NP
MDRV-P10
gE
β-actin
G
NIH/ 3T3 cell
WSN (hpi) 0 8 12 24
mNEAT1 (mNEAT1_1 + mNEAT1_2)
mNEAT1_2
NP
β-actin
H
mNEAT1 (mNEAT1_1 + mNEAT1_2)
mNEAT1_2
Relative lncRNA levels
p<0.0001
p=0.0008
p=0.0011
p=0.0007
p=0.0003
p=0.0011
WSN(hpi) 0 8 12 24 0 8 12 24
NIH/3T3 cell
I
L929 cell
PR8 (hpi) - +
mNEAT1 (mNEAT1_1 + mNEAT1_2)
mNEAT1_2
NP
β-actin
J
WT mice lung
WSN (hpi) 0 8 12 24
mNEAT1 (mNEAT1_1 + mNEAT1_2)
mNEAT1_2
NP
β-actin

Figure S2 NEAT1 expression is significantly induced by infections with several viruses

(A) Shown is a paradigm of the primers location of human NEAT1.

(B) 293T cells were infected with or without PR8 at the indicated MOIs for 16 h. RT-PCR was performed to determine the expression of human NEAT1 (NEAT1 and NEAT1_2). Shown are representative results from three independent experiments.

(C-E) RT-PCR was employed to detect the RNA levels of human NEAT1 (NEAT1 and NEAT1_2) in cells infected with or without CA04 (C), H3N2 (D), SeV (E), MDRV (E), or PRV (E) (MOI=1). Shown are representative results from three independent experiments.

(F-H) Mouse NIH/3T3 cells were infected with or without PR8 (F) or WSN (G and H) (MOI=1) for indicated time points. Expression of mouse NEAT1 (mNEAT1 and mNEAT1_2) was assessed by RT-PCR (F and G) and quantitative real-time PCR (H). Data are represented as mean ± SD from three independent experiments.

(I) RT-PCR was employed to detect the expression of mouse NEAT1 (mNEAT1 and mNEAT1_2) in PR8(MOI=1)-infected or mock-infected L929 cells. Shown are representative results from three independent experiments.

(J) WT C57BL/6J mice (6 weeks) were intranasally inoculated with WSN (5×10^4 PFU/mL) for indicated time point, lung tissues were collected and lysed, and RT-PCR was performed to analyze the levels of mouse NEAT1 (mNEAT1 and mNEAT1_2). Shown are representative results from three independent experiments.

Fig.S3

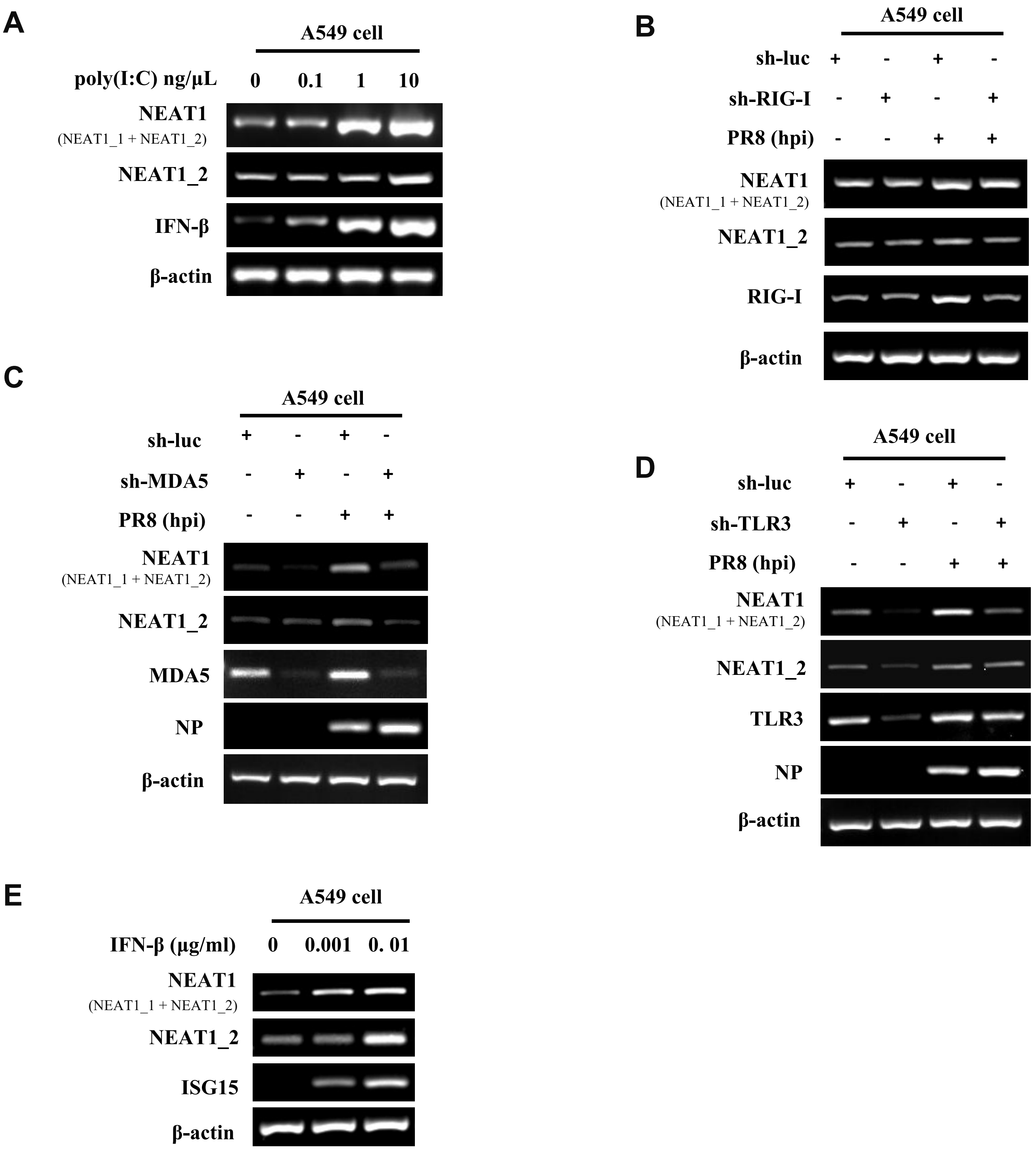
A
A549 cell
poly(I:C) ng/μL
0
0.1
1
10
NEAT1
(NEAT1_1 + NEAT1_2)
NEAT1_2
IFN-β
β-actin
B
A549 cell
sh-luc + - + -
sh-RIG-I - + - +
PR8 (hpi) - - + +
NEAT1
(NEAT1_1 + NEAT1_2)
NEAT1_2
RIG-I
β-actin
C
A549 cell
sh-luc + - + -
sh-MDA5 - + - +
PR8 (hpi) - - + +
NEAT1
(NEAT1_1 + NEAT1_2)
NEAT1_2
MDA5
NP
β-actin
D
A549 cell
sh-luc + - + -
sh-TLR3 - + - +
PR8 (hpi) - - + +
NEAT1
(NEAT1_1 + NEAT1_2)
NEAT1_2
TLR3
NP
β-actin
E
A549 cell
IFN-β (μg/ml)
0
0.001
0. 01
NEAT1
(NEAT1_1 + NEAT1_2)
NEAT1_2
ISG15
β-actin

Figure S3 Poly(I:C) and IFN-β can induce the expression of NEAT1, while RIG-I is not involved in regulating NEAT1 expression

(A) A549 cells were transfected with poly(I:C) at the indicated concentrations for 4 h. The levels of human NEAT1 (NEAT1 and NEAT1_2) were examined by RT-PCR. Shown are representative results from three independent experiments.

(B) RT-PCR was performed to detect the expression of human NEAT1 (NEAT1 and NEAT1_2) in PR8 (MOI=1)-infected or mock-infected RIG-I-knockdown A549 cells. Shown are representative results from three independent experiments.

(C and D) RT-PCR was applied to examine the expression of human NEAT1 (NEAT1 and NEAT1_2) in MDA5 (C)- or TLR3 (D)-knockdown A549 cells infected with or without PR8 (MOI=1) for 16 h. Shown are representative results from three independent experiments.

(E) A549 cells were treated with IFN-β at indicated concentrations for 2 h, and expression of human NEAT1 (NEAT1 and NEAT1_2) was examined by RT-PCR. Shown are representative results from three independent experiments.

Fig.S4

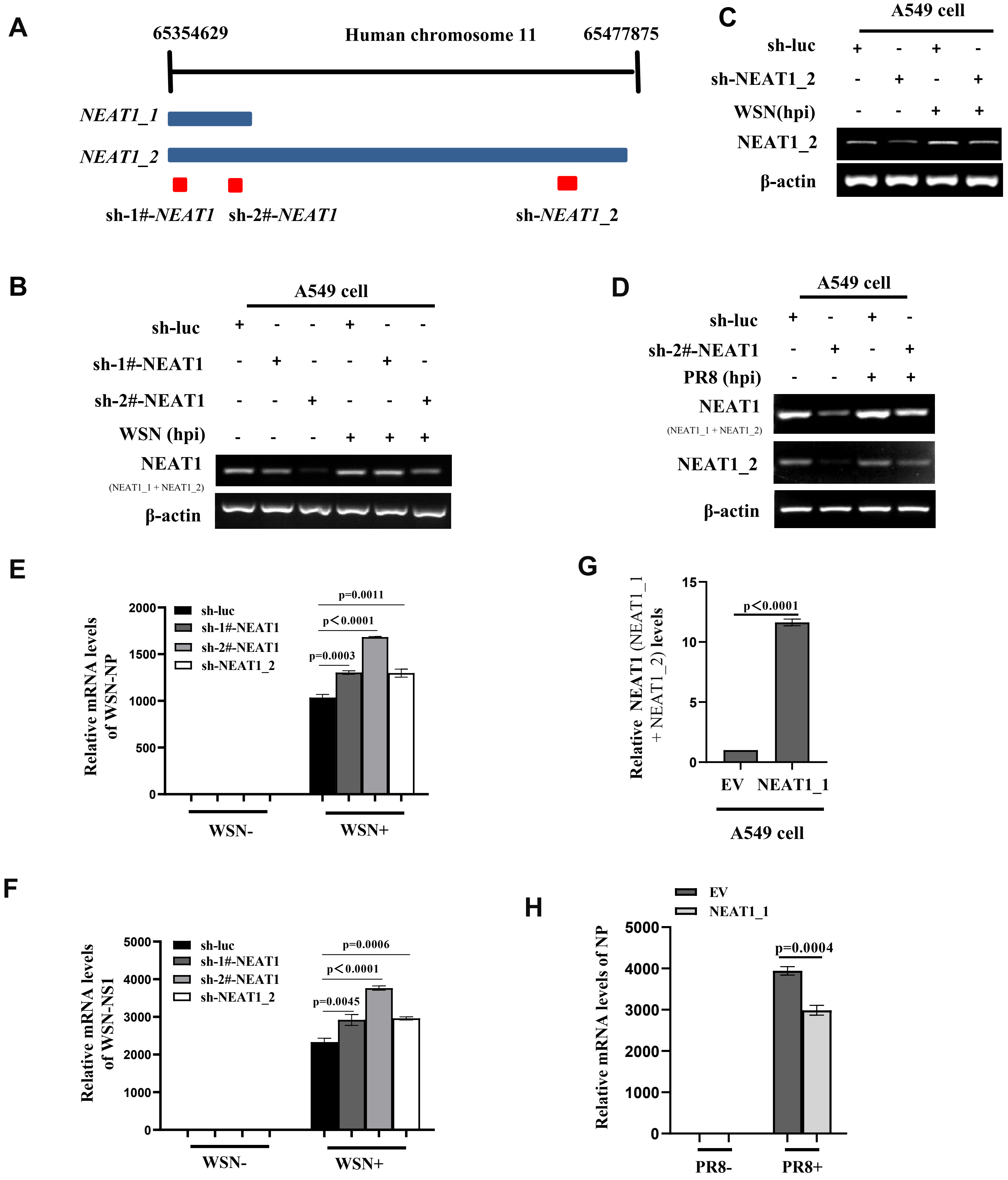
A
65354629
Human chromosome 11
65477875
NEAT1_1
NEAT1_2
sh-1#-NEAT1
sh-2#-NEAT1
sh-NEAT1_2
C
A549 cell
sh-luc
sh-NEAT1_2
WSN(hpi)
NEAT1_2
β-actin
B
A549 cell
sh-luc
sh-1#-NEAT1
sh-2#-NEAT1
WSN (hpi)
NEAT1
(NEAT1_1 + NEAT1_2)
β-actin
D
A549 cell
sh-luc
sh-2#-NEAT1
PR8 (hpi)
NEAT1
(NEAT1_1 + NEAT1_2)
NEAT1_2
β-actin
E
Relative mRNA levels of WSN-NP
sh-luc
sh-1#-NEAT1
sh-2#-NEAT1
sh-NEAT1_2
p=0.0011
p<0.0001
p=0.0003
WSN-
WSN+
G
Relative NEAT1 (NEAT1_1 + NEAT1_2) levels
p<0.0001
EV
NEAT1_1
A549 cell
F
Relative mRNA levels of WSN-NS1
sh-luc
sh-1#-NEAT1
sh-2#-NEAT1
sh-NEAT1_2
p=0.0006
p<0.0001
p=0.0045
WSN-
WSN+
H
EV
NEAT1_1
Relative mRNA levels of NP
p=0.0004
PR8-
PR8+

Figure S4 Altering NEAT1 expression has significant effects on the viral replication *in vitro*

(A) Shown is a paradigm of the shRNA location of human NEAT1.

(B and C) RT-PCR was utilized to examine the knockdown efficiency of shRNAs specifically targeting NEAT1 (sh-1#-NEAT1 and sh-2#-NEAT1) (B) or (sh-NEAT1_2) (C) in A549 cells. Shown are representative results from three independent experiments.

(D) The expression of human NEAT1_2 in sh-2#-NEAT1 A549 cells was examined by RT-PCR. Shown are representative results from three independent experiments.

(E and F) Quantitative real-time PCR assay was employed to detect the mRNA levels of viral NP (E) and NS1 (F) in NEAT1-knockdown A549 cells infected with or without WSN (MOI=1). Data are represented as mean ± SD from three independent experiments.

(G) The mRNA levels of NEAT1_1 in NEAT1_1-overexpressing A549 cells were detected by quantitative real-time PCR. Data are represented as mean ± SD from three independent experiments.

(H) Quantitative real-time PCR was performed to detect the mRNA levels of viral NP in NEAT1_1-overexpressing A549 cells or control A549 cells infected with or without PR8 (MOI=1). Data are represented as mean ± SD from three independent experiments.

Fig.S5

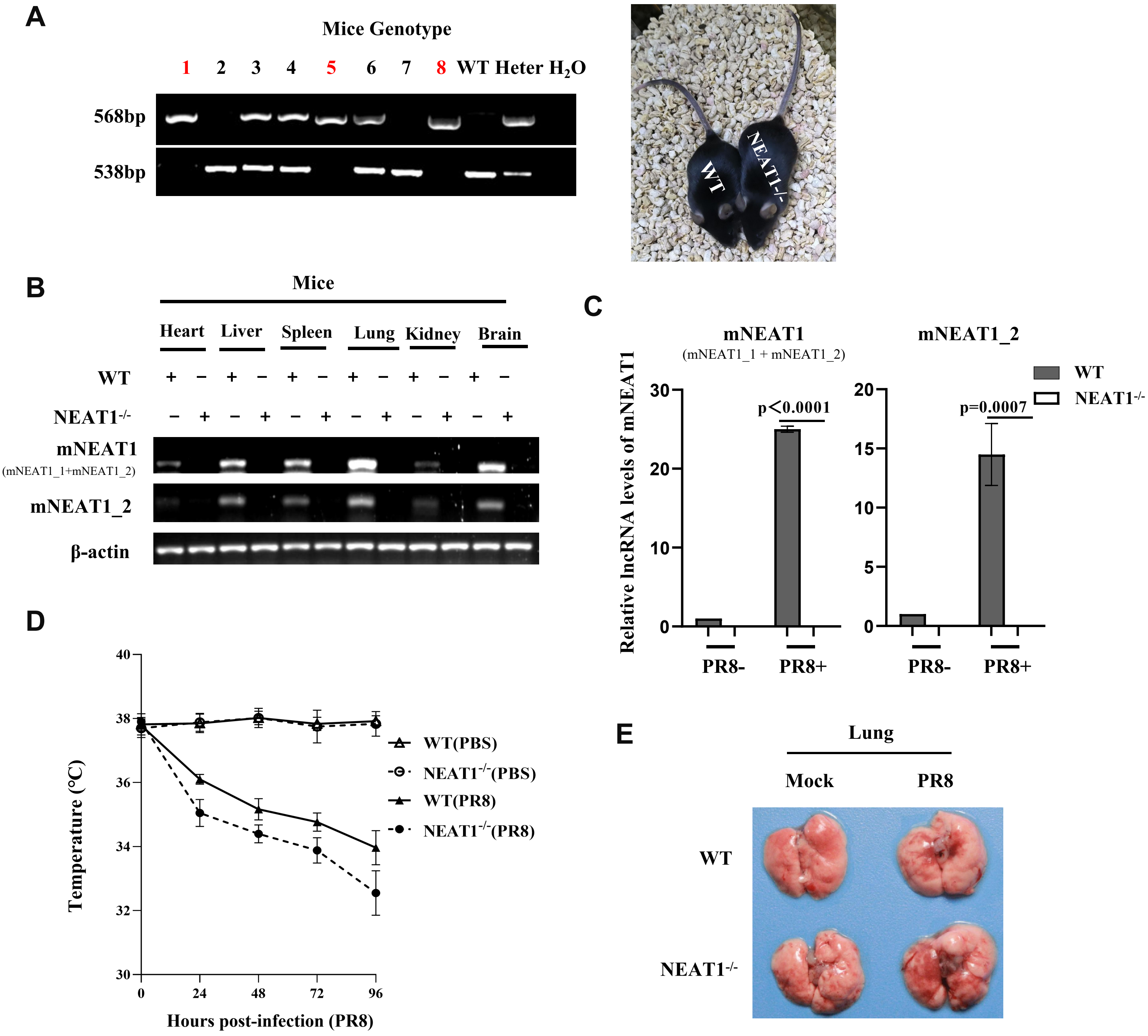

A
Mice Genotype
1 2 3 4 5 6 7 8 WT Heter H2O
568bp
538bp
WT
NEAT1-/-
B
Mice
Heart Liver Spleen Lung Kidney Brain
WT
NEAT1-/-
mNEAT1
(mNEAT1_1+mNEAT1_2)
mNEAT1_2
β-actin
C
mNEAT1
(mNEAT1_1 + mNEAT1_2)
mNEAT1_2
Relative lncRNA levels of mNEAT1
p<0.0001
p=0.0007
WT
NEAT1-/-
PR8-
PR8+
D
Temperature (°C)
Hours post-infection (PR8)
WT(PBS)
NEAT1-/-(PBS)
WT(PR8)
NEAT1-/-(PR8)
E
Lung
Mock
PR8
WT
NEAT1-/-

Figure S5 NEAT1 knockout mice are more susceptible to IAV infection

(A) Left: The genotype of C57BL/6J NEAT1 knockout ($NEAT1^{-/-}$) mice were determined by PCR of mouse tail DNA. Right: Photos of WT and $NEAT1^{-/-}$ mice are shown.

(B) The levels of mouse NEAT1 (mNEAT1 and mNEAT1_2) in different organs of $NEAT1^{-/-}$ mice and WT mice were measured by RT-PCR. Shown are representative results from three independent experiments.

(C) Quantitative real-time PCR was performed to analyze the expression of mouse NEAT1 (mNEAT1 and mNEAT1_2) in the lungs of $NEAT1^{-/-}$ mice and WT mice (6 weeks) infected with PR8 (5×10^4 PFU/mL) for 48 h. Data are represented as mean ± SD from three independent experiments.

(D) C57BL/6J WT mice and $NEAT1^{-/-}$ mice (6 weeks) were intranasally inoculated with PR8 (5×10^4PFU/mL). The body temperature change of mice were monitored.

(E) Shown are representative images of the lungs from WT and $NEAT1^{-/-}$ mice with or without PR8 infection.

Fig.S6

A

The sequence of chicken lncRNA-UP4 (TCONS_00098959) :

GATTTACTCATATTTTCTTGCAGTTAAGAAATAAAGTTTCTATCAAAAATTAGATAAAGCAAGTGCAAAGCGTGAG
CAGTTATTGACCTGGGAAGTTAGGTAAACATGGGTTTGCAATTACATCATCTTGTGATTTCTCAGGATCCACTTTAT
TCTCACAGAAGCTTCCCCAATGGTGCCATTCATGGCACAGCAACTGGAAAACAGGTCATTTTCAGTAAGACTGGAA
GGTCATCTGTACTTCTGCCTTCATCCTAAATGATCTCCCATCTCACAAATTCTGTTTTCTCTTATAGCCCTGTCCCAG
GCCATTGAGCCATGTGATCAGGAGGGATATGAGACTTGGAATTTGAGGAACATCTTTGAACTTCATCATGTTTGTT
AACACGTGCTAATGTGGGATCCACGGAGGAGGCTTAAATTGGACATAAGGAAGAATCTCTTCATGAAGAGGTTGA
TCAGGCACTGGAACAGGCTGACCAGGGAAATGGTGGTGTCCCCATCCTTGGAGGTGTTTAAGAGATGTGTGGATG
TAGCACTGAGGGACATGGTAAGTGGTGGGACTCAGTAGCCCAGGTTGATGGCTGGATTAAGGGTCTTTTCCAACCT
AATTGAGTGCATGATTCTATGGAGGTGTCTGTGGAGGAGCTGCAGATAAAATGGTGGAAACTCAGTGAATGCATC
AGCATTTCTCTTCCCTCCAGGAAAAACTTGAAGTTGACCTTCCCACGCAGTAAGCAGTGATCTTTTCTATCTCTTAC
ATCCCAAAATGTAGTAACATTTACACCTTTACTGGAATGCTGGACAGCCTAGGCAGCCACCAGCTGAAGGGTGTCT
TCTGGGAATGTGGATGTTCCTCAGTCTGTTGTGTGAAATTTCACTGCTCTGCAGTTTAGATGCCTCCTGGAATTCAA
GGAGTTCAATCCAAAGAGTGAGTTTGTTTAGCTGAAGTGTACACAGTAACTAAGAATGATCTTAGGCAATCCATCA
CCTGCTGCTGGTAGAGGTGAGATAAACATTTCTGGTTTGTGTTTGTTTTGTTCCCTGCTACTGTCACAGTAATACAG
TGCTCAAAGGGTCCAACTCGGGATCCCCGTTGAGGTCTCCTTTGTGAATCAGAAGTAGTGTGATTCATACTGTACTT
GTGAATAGATTGTGCTTTTCATAGCTGCTATCTAAGCCCTTATCTGCAGTAAAGAGGGAGCAAGATCGAGATCCTT
GGTAGTAAACCCCTCATGCATTACCTCGGTCAGAGAGCTCTATATTTTTAAAGACACTTACTGAATTCTTACTGAAG
ATACTCTAGAATTTCGTCAAGACCAACAGATTCAAATACCTCATTTCCTTCTCAGAGTCACGTGCGTACAAGACTG
GAGCCCAGTAGCTTTGAGACAGATTGTTCCATATTACTCAGAGAAAGCAAAATGATTTAGTCATTCTCATGGCTTT
CCCATAGCTGTCACAGACATAGTAGGTACCAAAATGCTCTCTCACCAAGTTGTCACTAAGAAAATCTCTGCCAAGG
TAAGCTGTGAAAAAGCCAACCAGATATTTGTATGTTGGTTCTATGATTCTATGTCTCTTAGACCATGTGCAGGACA
ACTAAAGCACTATCTTCTGTCTTGCAAGAAAATCCCACTCACCACAGACTTCTTGAAAATGACCACTTTGAGGTCT
CTTCATTCTTTCCTGAGGTGTTGCACAATTTGAGCCTATGGTTTTACCATATTGGGCAAAGCAATTGCACTTTGCTTT
GTTTTGAGCCTCTGAGATGCAGGCTGTGAGCTGACTGCTCACTGTTTTGGCATCCCCAGCAGTGGAATGTCTGTGTG
GATGATCAGAAGATCGCTCAGAGGAAGGGAAGAGTTTACTGACATCCTTATCCACATTCCAGAGCATACCTTTCAT
CTCCTCATTGTGCTGCTCCAGAGAGAGTAATTAATGTTCCACCGTTCACACTGGAAGAATAAGGCTCAAGGGAGCC
CATCCTTTGTGCGTGCTTTGAGCTGAAATTCCTGTAAGTTTGGGTATATGTGGATGTTCACAGCCACATCAAAATAG
GCGATACAGCTAAGGAATATTTCAGTGGTTTGGTACTCAGTCTAAGGACTTATTTTTAGCTCTGCATTGCTCAGTGA
CTTGCATTCATGCTCTGTGACAAATGGATTAGATCCCAAGAGTCATGACATCACTGTAACAAGACGTCAATTCCTC

B

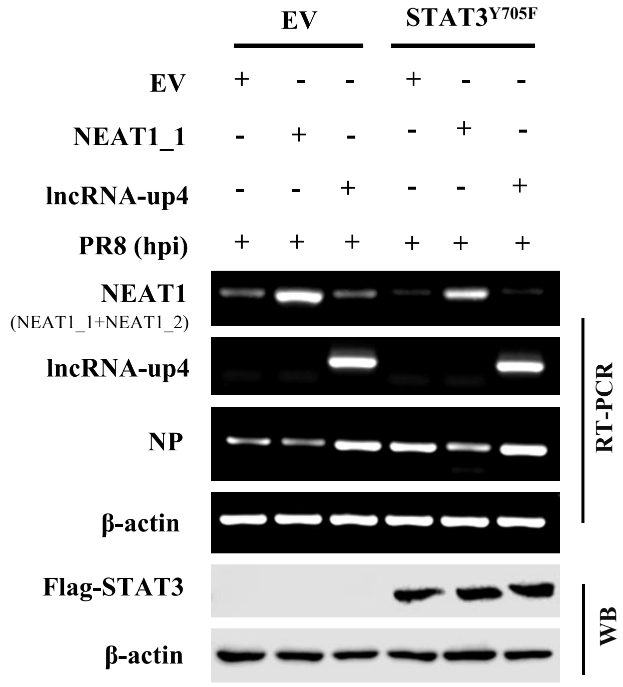

Figure S6 Activated STAT3 suppresses IAV replication through positively regulating the expression of NEAT1

(A) The sequence of chicken lncRNA-UP4 (TCONS_00098959). Through analysis of RNA sequencing data from H9N2 avian influenza virus-infected chicken DF-1 cells (GEO accession no: GSE279270), it was observed that lncRNA-up4 was significantly upregulated during H9N2 infection.

(B) A549 cell lines stably expressing Flag-$STAT3^{Y705F}$ ($STAT3^{Y705F}$), or empty vector (EV) were transfected with EV, NEAT1_1, or chicken lncRNA-up4, followed by infection with PR8 virus (MOI=1) for 16 h. The mRNA levels of viral NP in the cells were examined by RT-PCR. Shown are representative results from three independent experiments.

Fig.S7

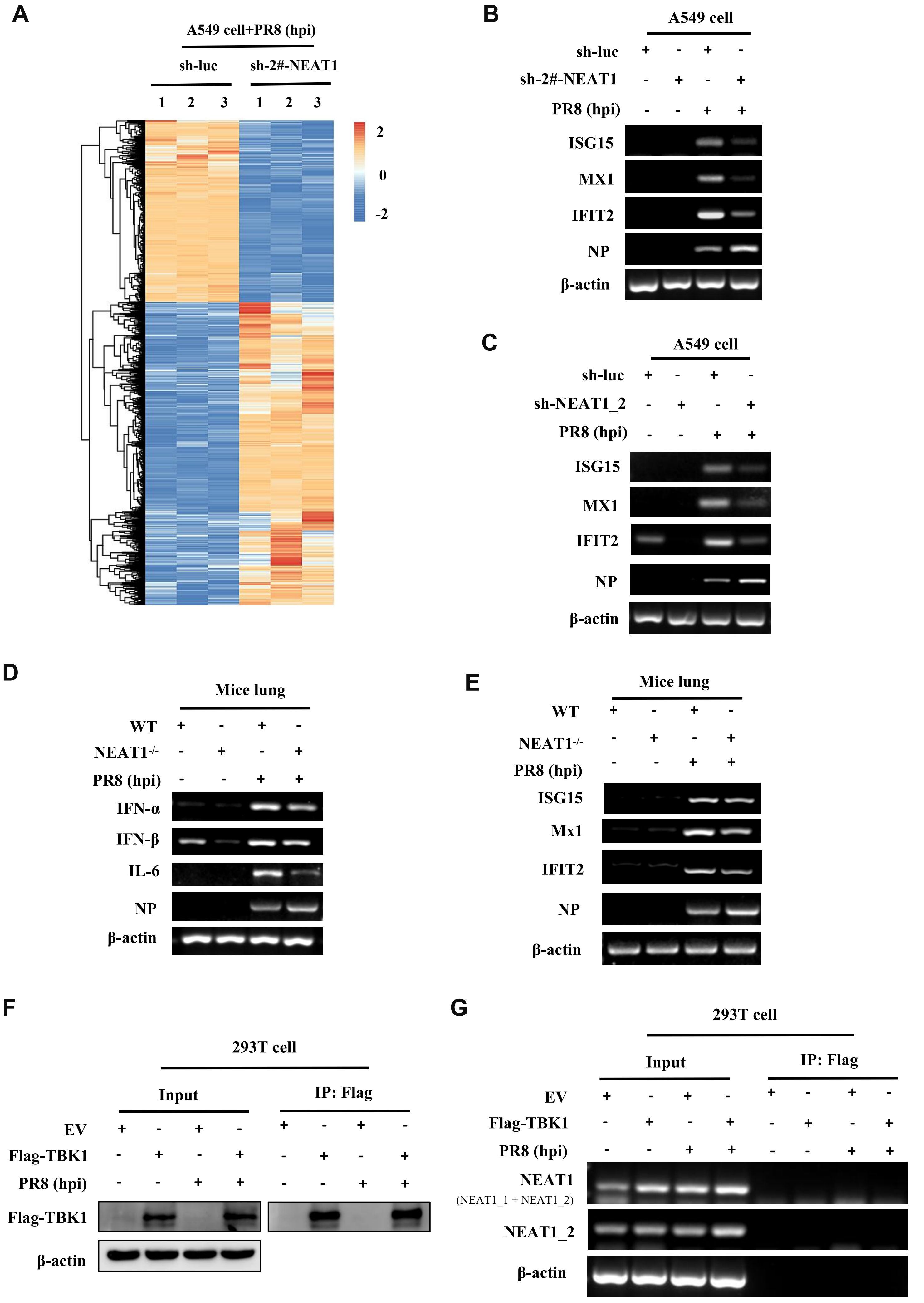
A
A549 cell+PR8 (hpi)
sh-luc
sh-2#-NEAT1
1 2 3 1 2 3
2
0
-2
B
A549 cell
sh-luc
sh-2#-NEAT1
PR8 (hpi)
ISG15
MX1
IFIT2
NP
β-actin
C
A549 cell
sh-luc
sh-NEAT1_2
PR8 (hpi)
ISG15
MX1
IFIT2
NP
β-actin
D
Mice lung
WT
NEAT1-/-
PR8 (hpi)
IFN-α
IFN-β
IL-6
NP
β-actin
E
Mice lung
WT
NEAT1-/-
PR8 (hpi)
ISG15
Mx1
IFIT2
NP
β-actin
F
293T cell
Input
IP: Flag
EV
Flag-TBK1
PR8 (hpi)
Flag-TBK1
β-actin
G
293T cell
Input
IP: Flag
EV
Flag-TBK1
PR8 (hpi)
NEAT1
(NEAT1_1 + NEAT1_2)
NEAT1_2
β-actin

Figure S7 NEAT1 is involved in regulation of innate antiviral responses

(A) Control and sh-2#-NEAT1 based knockdown A549 cells were infected with PR8 for 16 h. The differentially expressed genes were detected by RNA-Seq (fold change >2, P <0.05).

(B and C) Control and NEAT1 (2#-NEAT1 or NEAT1_2)-knockdown A549 cells were infected with or without PR8 for 16 h. The mRNA levels of ISG15, MX1, and IFIT2 were examined by RT-PCR. Shown are representative data from three independent experiments.

(D and E) RT-PCR was performed to analyze the levels of IFN-α (D), IFN-β (D), IL-6 (D) or ISGs (E) in the lungs from WT mice and $NEAT1^{-/-}$ mice (6 weeks) at 48 h post PR8 (5×10^4 PFU/mL) virus infection. Shown are representative results from three independent experiments.

(F and G) EV and Flag-TBK1 overexpressing 293T cells were infected with PR8 (MOI = 1) for 16 h, and then subjected to RNA immunoprecipitation (RIP) assays using Flag-antibody-beads. β-actin served as the negative control. Western blotting (F) and RT-PCR (G) were repeated independently three times with similar results.

Fig.S8

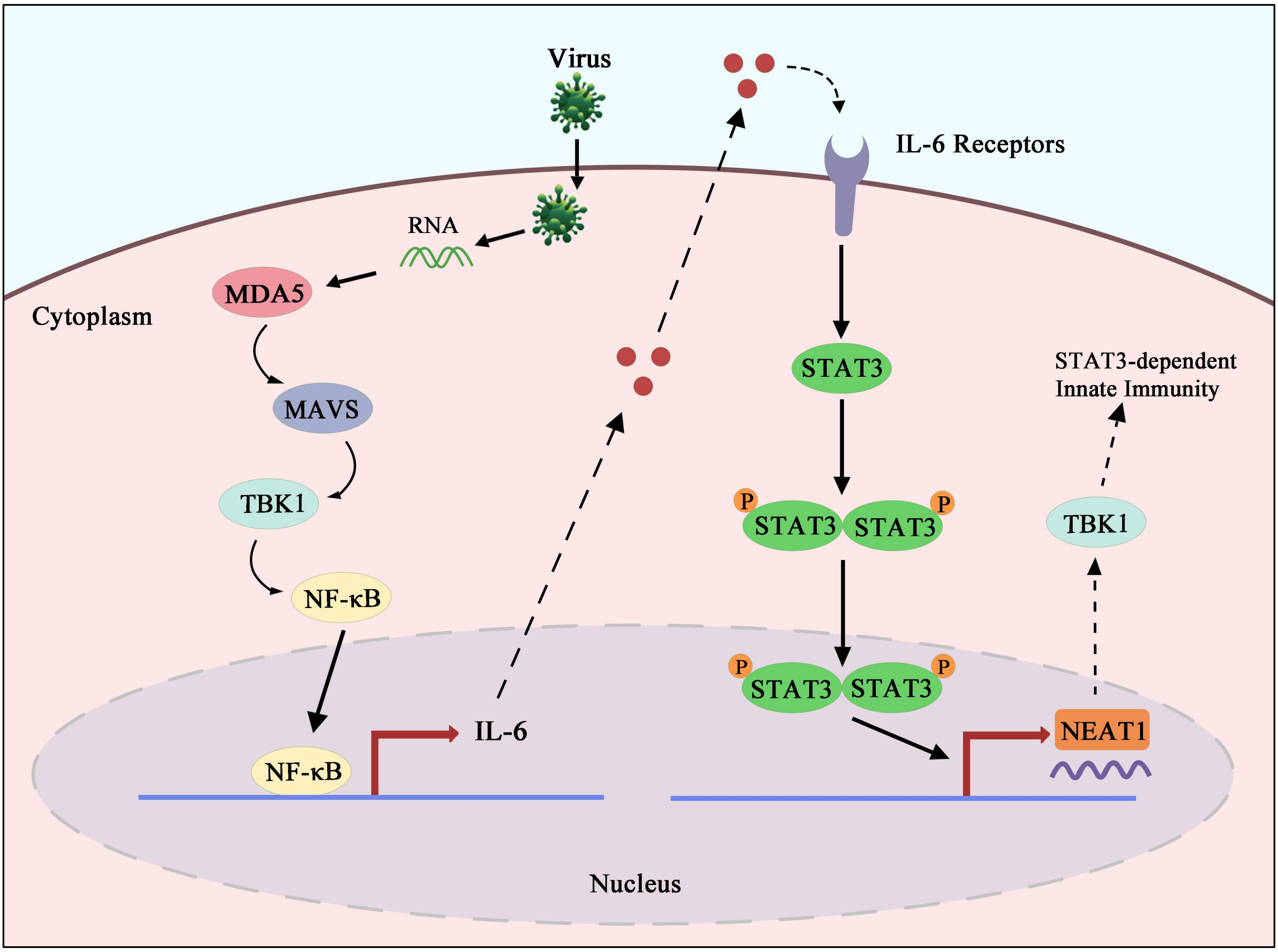

Virus
IL-6 Receptors
RNA
MDA5
Cytoplasm
MAVS
TBK1
NF-κB
STAT3
STAT3-dependent
Innate Immunity
P
STAT3
STAT3
P
TBK1
P
STAT3
STAT3
P
NF-κB
IL-6
NEAT1
Nucleus

Figure S8 Schematic diagram of NEAT1 induction and function during the influenza A virus infection

Upon recognition of viral components by MDA5, the adapter protein MAVS promotes IL-6 production through the downstream TBK1 and NF-κB signaling pathways. The subsequent binding of IL-6 to its receptor triggers STAT3 activation, leading to the upregulation of NEAT1 expression. The increased levels of NEAT1, in turn, enhance STAT3-mediated antiviral innate immunity by facilitating TBK1 activation.
